# Multivalvular Involvement in Acute Heart Failure: Associations with One-Year Outcomes and the Right-Heart Phenotype

**DOI:** 10.3390/medsci14050564

**Published:** 2026-09-12

**Authors:** Georgios Aletras, Stylianos Fiflis, Theodora Georgopoulou, Georgia Halkiadaki, Yannis Pantazis, Konstantinos Stylianou, Michalis Hamilos, Emmanuel Foukarakis

**Affiliations:** 1 Department of Cardiology, Venizelio General Hospital of Heraklion, 71409 Heraklion, Greece; stylianosfiflis@yahoo.com (S.F.); theodgeo7@gmail.com (T.G.); mfouk@hotmail.com (E.F.); 2 School of Medicine, University of Crete, 70013 Heraklion, Greece; kstylianu@med.uoc.gr (K.S.); mchamilos@uoc.gr (M.H.); 3 Department of Biochemistry, Venizelio General Hospital of Heraklion, 71409 Heraklion, Greece; giouliha@yahoo.gr; 4 Institution of Applied and Computational Mathematics, Foundation of Research and Technology-Hellas, 70013 Heraklion, Greece; pantazis@iacm.forth.gr; 5 Department of Nephrology, University General Hospital of Heraklion, 71500 Heraklion, Greece; 6 Department of Cardiology, University General Hospital of Heraklion, 71500 Heraklion, Greece

**Keywords:** acute heart failure, valvular heart disease, multivalvular disease, mitral regurgitation, tricuspid regurgitation, prognosis, mortality

## Abstract

**Background:** Valvular heart disease frequently accompanies acute heart failure (AHF), yet most studies address single valve lesions, and the prognostic weight of concurrent multivalvular involvement is less well defined. We examined the prevalence of single- and multi-valve disease and its relationship with one-year mortality. **Methods:** We analyzed 530 consecutive patients enrolled in a prospective single-center AHF registry between February 2023 and June 2025 and followed for 12 months. Aortic, mitral and tricuspid disease was graded according to European Society of Cardiology/European Association of Cardiovascular Imaging (ESC/EACVI) criteria, a valve being considered involved when it carried at least moderate stenosis and/or regurgitation; multivalvular disease was defined as involvement of two or more valves. The primary endpoint was one-year all-cause mortality. Cox regression was adjusted for admission log N-terminal pro-B-type natriuretic peptide (NT-proBNP), frailty (Clinical Frailty Scale ≥ 5) and age. In-hospital acute kidney injury was examined descriptively but was not entered into the models, because it is ascertained after admission. **Results:** Overall, 71.3% of patients had at least one significantly diseased valve—mitral in 44.3%, tricuspid in 43.8% and aortic in 27.5%—and 34.9% had multivalvular involvement. One-year mortality rose stepwise with the number of valves involved: 19.7%, 24.9%, 36.3% and 54.0% for zero, one, two and three valves, respectively (log-rank *p* < 0.001), corresponding to a crude hazard ratio (HR) of 1.51 (95% confidence interval [CI] 1.28–1.78) per additional valve. Multivalvular versus single or no valve involvement carried a crude HR of 2.07 (1.51–2.84) and remained associated with mortality after adjustment (HR 1.48, 95% CI 1.06–2.07, *p* = 0.020), as did each additional valve (HR 1.22, 1.03–1.45, *p* = 0.023). Adjustment for clinically manifest right heart failure (HF) attenuated the association with mortality (HR 1.15, 95% CI 0.96–1.37), whereas the association with the triple composite remained significant (HR 1.19, 95% CI 1.03–1.36). Propensity-score adjustment excluding right HF retained the association with mortality (HR 1.43, 95% CI 1.02–2.00), whereas inclusion of right HF attenuated it (HR 1.33, 0.94–1.87). The association was not detectable in the 304 patients without clinically manifest right HF (HR 1.06, 0.80–1.41, *p* = 0.69). In a post-discharge sensitivity analysis, the adjusted associations were directionally similar but did not reach statistical significance. **Conclusions:** In hospitalized AHF, multivalvular involvement was common, followed a predominantly mitral–tricuspid pattern, and identified an older, frailer, more congested phenotype with substantial right-sided and chronic renal involvement. Valve burden showed a graded association with one-year mortality that persisted after adjustment for baseline prognostic characteristics, but was attenuated once right-sided involvement was taken into account, whether by direct adjustment or by propensity methods; the association was retained when right HF was omitted from the propensity model. Because right-sided variables lie downstream of significant tricuspid disease, this attenuation is what adjustment for an intermediate is expected to produce and does not establish absence of prognostic value. Cumulative valve burden is therefore best interpreted as a powerful phenotypic marker of a more advanced HF phenotype with prominent right-heart involvement, rather than as an independent causal determinant of mortality.

## 1. Introduction

Heart failure (HF) affects more than 64 million people worldwide and remains a leading cause of hospitalization and death in older adults, with a prevalence that continues to rise as populations age [1]. Valvular heart disease is highly prevalent among patients admitted with acute heart failure (AHF), where it contributes both to the episode of decompensation and to the subsequent adverse outcome [2]. Significant valve lesions are common in older community populations [3], and in the acute setting their presence may aggravate the clinical picture and signal diminished reserve to compensate for superimposed insults such as ischemia, arrhythmia, infection or anemia. Valvular lesions frequently also represent a consequence of ventricular and atrial remodeling and a marker of advanced cardiac disease. Contemporary guidelines and the bulk of the outcome literature are nevertheless organized around individual valve lesions—aortic stenosis, mitral regurgitation, tricuspid regurgitation—each considered and graded in isolation [4].

In everyday practice, however, valve lesions rarely occur alone. Aging, atrial fibrillation (AF), pressure and volume overload and annular dilation frequently produce disease across more than one valve simultaneously, and such multivalvular involvement is increasingly recognized as a distinct and challenging entity whose hemodynamic interactions complicate assessment and whose management is poorly served by lesion-specific evidence [5,6]. Recent data in patients with moderate aortic regurgitation suggest that concomitant moderate valve lesions may confer a risk comparable to that of isolated significant disease [7,8]. Dedicated guidance nonetheless remains scarce: recommendations rest largely on small retrospective series and expert consensus, and patients with multiple concurrent lesions are systematically underrepresented in randomized trials, so that both severity assessment and clinical decision-making in this population remain poorly standardized [2,4,9]. Whether the simple accumulation of affected valves carries prognostic meaning in AHF—over and above the recognized drivers of outcome such as congestion, frailty and renal dysfunction—has received comparatively little attention.

We therefore used a prospective single-center AHF registry to (i) describe the prevalence of significant single- and multi-valve disease in patients hospitalized for AHF, (ii) characterize the clinical phenotype associated with multivalvular involvement, and (iii) determine whether the number of significantly diseased valves is associated with one-year all-cause mortality, both crudely and after adjustment for established prognostic markers.

## 2. Materials and Methods

### 2.1. Study Design and Population

This prospective, observational, single-center study was conducted in the Department of Cardiology of Venizelio General Hospital, Heraklion, Crete, Greece. Consecutive adults admitted between February 2023 and June 2025 with a primary diagnosis of AHF were enrolled and followed for 12 months. AHF was diagnosed, in accordance with the 2021 European Society of Cardiology (ESC) Heart Failure Guidelines [10], by the combination of typical symptoms and signs (dyspnea, orthopnea, raised jugular venous pressure, peripheral edema, pulmonary crackles) with supporting objective evidence (elevated natriuretic peptides, structural cardiac abnormality, a dilated inferior vena cava or pulmonary B-lines on ultrasound). Patients under 18 years of age, those already receiving renal replacement therapy (RRT), and those admitted primarily for an acute coronary syndrome without a dominant AHF presentation were not eligible. Only the first eligible admission for each patient during the enrolment period was included, so that each of the 530 patients in the analysis cohort contributes a single index hospitalization. Of 592 patients assessed for eligibility, 62 met one or more exclusion criteria and were not enrolled, leaving a final analysis cohort of 530 patients with complete one-year follow-up and no loss to follow-up. Each patient was followed for 12 months, the last follow-up contact taking place in June 2026. Vital status at 12 months was ascertained in all 530 patients through outpatient review, structured telephone contact and the hospital record system. Written informed consent was obtained from all participants; the study was approved by the Institutional Ethics Committee of Venizelio General Hospital of Heraklion on 5 August 2022 (protocol No. 15) and complied with the Declaration of Helsinki.

### 2.2. Echocardiographic Assessment and Valve Definitions

Transthoracic echocardiography was performed in all patients during the index hospitalization, after initial clinical stabilization, and interpreted by two separate experienced cardiologists using standard commercially available equipment. Studies were performed at a median of three days after admission (interquartile range [IQR] 2–5 days). All patients, including the 27 who died during the index hospitalization, underwent echocardiography before any study endpoint occurred, so no outcome preceded determination of valve burden. Studies were performed at this time point, that is, after decongestive therapy had generally been initiated, which reduces the likelihood that loading-dependent regurgitation is overestimated at the height of congestion; the timing was not, however, standardized to a predefined euvolemic state. The severity of aortic stenosis, aortic regurgitation, mitral regurgitation, mitral stenosis and tricuspid regurgitation was graded as absent/mild, moderate or severe according to contemporary ESC/European Association for Cardio-Thoracic Surgery (EACTS) and European Association of Cardiovascular Imaging (EACVI) recommendations [4,11,12], integrating qualitative, semiquantitative and quantitative parameters as appropriate for each lesion. Studies were not re-adjudicated by an independent core laboratory, and formal interobserver reproducibility was not assessed.

For the present analysis, a valve was classified as “involved” when it carried at least moderate stenosis and/or regurgitation. The aortic, mitral and tricuspid valves were each treated as a single anatomical unit, so that mixed disease of one valve (for example combined moderate aortic stenosis and aortic regurgitation) counted as involvement of that valve only, not as two lesions. The number of involved valves was summed for each patient (0–3), and multivalvular disease was defined as involvement of two or more valves. The pulmonary valve was not routinely quantified and was not included. This valve-based definition is fully reproducible from the individual lesion gradings and is summarized in Supplementary Table S1; because no consensus definition exists, alternative and stricter criteria were examined in sensitivity analyses.

In patients with a previously implanted valve prosthesis, the prosthetic valve was classified as involved only when prosthetic dysfunction was documented—that is, when prosthetic stenosis or regurgitation (transvalvular or paravalvular) of at least moderate degree was present. A normally functioning prosthesis was therefore not counted as valve involvement, so that the valve-burden variable reflects hemodynamically significant valve disease at the time of the index admission rather than the history of valve intervention itself.

### 2.3. Definitions

Coronary artery disease was considered present where invasive or computed-tomography coronary angiography demonstrated at least 50% stenosis of one or more epicardial vessels, or where there was equivalent evidence in the form of prior myocardial infarction or previous revascularization. Because coronary anatomy is not systematically assessed in every patient admitted with AHF, coronary status was recorded in three categories—documented, absent, or not assessed—rather than being dichotomized, and patients without any coronary evaluation were not assumed to be free of disease. An ischemic etiology of HF was assigned where coronary disease was judged the dominant cause of ventricular dysfunction; this variable records the primary etiology of the HF syndrome and is therefore not interchangeable with the presence of coronary artery disease.

Chronic kidney disease (CKD) was defined as an estimated glomerular filtration rate (eGFR) below 60 mL/min/1.73 m^2^ persisting for at least three months, and was staged according to the Kidney Disease: Improving Global Outcomes (KDIGO) 2012 classification (stage 1, ≥90; stage 2, 60–89; stage 3a, 45–59; stage 3b, 30–44; stage 4, 15–29 mL/min/1.73 m^2^). Baseline creatinine and the derived eGFR were taken from stable outpatient values in the year before admission, rather than the presentation value (distorted by acute congestion); where several were available, the most representative stable value was selected clinically, excluding measurements during intercurrent illness. Some measurement error in this estimate therefore cannot be excluded. Acute kidney injury (AKI) was defined by KDIGO criteria as a rise in serum creatinine of equal or more than 0.3 mg/dL or equal to more than 1.5 times the baseline value during hospitalization. Right HF was defined by the coexistence of at least two of the following: clinical systemic venous congestion without a predominant left-sided explanation; echocardiographic right ventricular (RV) dilation or systolic dysfunction (tricuspid annular plane systolic excursion [TAPSE] below 17 mm and/or tricuspid annular S′ below 9.5 cm/s); and elevated right-sided pressures, taken as an estimated pulmonary artery systolic pressure (PASP) above 35 mmHg or an intermediate-to-high echocardiographic probability of pulmonary hypertension graded according to the 2022 ESC/European Respiratory Society (ERS) criteria. AF was recorded where documented on electrocardiography or ambulatory monitoring, whether paroxysmal, persistent or permanent. De novo HF denoted a first presentation in a patient without previously known HF, as distinct from acute-on-chronic decompensation. Functional status was graded by New York Heart Association (NYHA) class referable to the patient’s stable condition before decompensation, in the same manner as the Clinical Frailty Scale (CFS).

Arterial hypertension, diabetes mellitus and airway disease were recorded on the basis of a prior physician diagnosis or of ongoing disease-specific treatment. Left and right ventricular dilation were defined by chamber dimensions indexed to body surface area exceeding contemporary guideline thresholds for age and sex.

### 2.4. Covariates, Endpoints and Follow-Up

Baseline demographic, clinical, laboratory and echocardiographic data were recorded at admission, together with the comorbidities defined above. Frailty was assessed with the CFS [13] referable to the patient’s pre-decompensation status, a score of 5 or more defining frailty, and N-terminal pro–B-type natriuretic peptide (NT-proBNP) was measured on admission. Patients were followed for 12 months through outpatient review or structured telephone contact.

The primary endpoint was one-year all-cause mortality. Two composite endpoints were additionally analyzed over the same period: a cardiorenal composite of death or RRT, and a triple composite of death, AHF rehospitalization or RRT. Composite endpoints were analyzed as time to the first occurrence of any component, ascertained over the full cohort. Non-fatal events were ascertained at scheduled follow-up checkpoints at 3, 6 and 12 months, with event dates recorded to the week. By design, ascertainment of subsequent non-fatal events ceased once a patient reached a terminal or component event: death was treated as a terminal event, and initiation of RRT, being itself a component of both composites, closed follow-up for the rehospitalization component. Composite event status was therefore determinate in all 530 patients, and the blank rehospitalization fields observed in patients who died or commenced RRT reflect this convention rather than loss to follow-up. For the triple composite, the order of events was reconstructed from the weekly event times: where an AHF rehospitalization preceded death or terminal RRT, the composite was dated to that rehospitalization rather than to death. This recovered 57 interval rehospitalizations that a checkpoint-based rule would otherwise have anchored to death. The total number of patients with a triple-composite event (258) is unchanged by this rule; only the timing of the first event differs. The renal endpoint was defined as initiation of RRT that remained necessary at the time of the last observation—at discharge, at a subsequent follow-up checkpoint, or at death. Temporary in-hospital dialysis followed by complete renal recovery was not counted. RRT was initiated during the index hospitalization or subsequent follow-up in 24 patients; one required temporary in-hospital dialysis, recovered renal function and needed no further treatment, leaving 23 patients (4.3%) meeting the renal endpoint—13 of whom subsequently died and 10 of whom remained alive on treatment at 12 months. Of these 23, 8 died during the index hospitalization and 15 survived to discharge. The cardiorenal composite of death or RRT therefore comprises 164 patients (154 deaths and 10 surviving patients on RRT).

### 2.5. Statistical Analysis

Continuous variables are summarized as median (IQR) after assessment of distribution and compared with the Mann–Whitney U test; categorical variables are presented as n (%) and compared with the χ^2^ test, or Fisher’s exact test where expected cell counts were small. No imputation was performed, and the available denominator is reported wherever data were incomplete. Missing data were confined to NT-proBNP (11, 2.1%), E/e′ (11) and single values for five other echocardiographic variables; all other variables were complete. Multivariable models were fitted on complete cases (n = 519 for models including NT-proBNP).

The primary analysis treated valve burden in two complementary ways. The number of involved valves (0–3) was examined as an ordered exposure, with one-year mortality reported for each stratum and time-to-event compared by Kaplan–Meier estimation with the log-rank test across all four groups; it was then modeled continuously as a single term, yielding an average hazard ratio (HR) per additional involved valve. In parallel, multivalvular disease was analyzed as the dichotomy of two or more involved valves versus single or no involvement, which is the form in which the entity is usually reported. Both are presented because dichotomization discards information while the dichotomy retains comparability with the existing literature.

Multivariable Cox proportional-hazards models were used to determine whether any association was independent of established determinants of outcome in AHF. Covariates were selected a priori on the basis of prior evidence rather than by data-driven procedures, and were deliberately restricted to three—admission log-transformed NT-proBNP as an integrated index of congestion and wall stress, frailty (CFS ≥ 5) as a measure of global physiological reserve, and age—so that the number of events per variable remained comfortably above the conventional minimum of ten. Variables ascertained during the index hospitalization, including in-hospital renal deterioration, were examined descriptively but were not entered into models anchored at admission, since they do not represent baseline covariates. Unlike in-hospital complications, valve burden was determined before any study endpoint occurred and was therefore treated as the index exposure for the complete 12-month observation period; a post-discharge sensitivity analysis beginning at discharge was performed. Because follow-up ended 12 months after admission, the post-discharge observation period varied according to length of stay, and survivors were censored at their individual end of follow-up. NT-proBNP was log-transformed because of its right-skewed distribution. Ties were handled by the Efron approximation, and confidence intervals (CI) were derived from the observed information matrix. Collinearity among model terms was assessed by variance inflation factors, all of which were below 1.5. Because congestion and frailty may partly lie on the pathway linking multivalvular disease to adverse outcome, the adjusted estimates should be interpreted as the association remaining after accounting for these accompanying clinical characteristics, rather than as the total prognostic association of valve burden.

Renal variables were examined descriptively across strata of valve burden, comprising baseline eGFR, KDIGO CKD stage, and in-hospital AKI, in order to distinguish any association with chronic renal impairment from one with acute in-hospital renal deterioration. Isolated RRT was not modeled separately because of the small number of renal events and the competing risk of death, which would make a conventional cause-specific analysis imprecise and potentially difficult to interpret.

Several model checks and sensitivity analyses were performed. First, an alternative severity-weighted score—summing each valve’s grade (0 absent/mild, 1 moderate, 2 severe; range 0–6), taking the higher grade where a valve carried both stenosis and regurgitation—was compared with the count in otherwise identical models. Second, the assumption that valve count acts linearly on the log-hazard scale was tested by comparing the continuous term with a four-level categorical parameterization (0, 1, 2, 3 valves) using a likelihood-ratio test. Third, the proportional-hazards assumption was examined for every term in the primary model using scaled Schoenfeld residuals and was satisfied throughout (all *p* > 0.12). Fourth, an expanded adjustment model additionally including sex, AF, left ventricular ejection fraction (LVEF), baseline eGFR and de novo versus acute-on-chronic status was fitted to assess residual confounding. Fifth, because right-sided involvement is closely bound up with tricuspid disease, right-sided variables were added to the primary model individually, and the primary model was refitted separately in patients with and without clinically manifest right HF. Sixth, the contribution of each anatomical valve was examined by entering the three valves simultaneously in an otherwise identical model, and baseline eGFR was added to the primary model to test whether any association simply reflected pre-existing renal impairment. Effect modification by CKD and by LVEF phenotype was assessed by multiplicative interaction terms, with estimates additionally reported within strata. The two composite endpoints were modeled with the same covariate set as the primary mortality analysis.

Because definitions of multivalvular disease are not standardized, three sensitivity analyses applied progressively stricter criteria to the same cohort: involvement of two or more valves each carrying severe disease; at least one severe lesion together with at least moderate disease of a second valve; and a left-sided definition analogous to that used in European surveys, namely severe aortic or mitral disease accompanied by at least moderate disease of the other left-sided valve. Each was analyzed with the same crude and adjusted models as the primary exposure, the intention being to establish whether the direction and approximate magnitude of any association were preserved rather than to identify an optimal threshold. These sensitivity and subgroup analyses were specified during the analysis phase rather than in a registered protocol and are therefore described as sensitivity rather than pre-specified analyses. A two-sided *p* < 0.05 was considered statistically significant; no formal correction for multiple comparisons was applied, and secondary results are interpreted as supportive rather than confirmatory. Analyses were performed in Python 3.12 using lifelines 0.27.8 (time-to-event analyses), statsmodels 0.14.1 (logistic regression), and pandas 2.2.1, NumPy 1.26.4, SciPy 1.12.0 and Matplotlib 3.8.3 for data management, computation and graphics (Python Software Foundation, Wilmington, DE, USA; open-source libraries).

## 3. Results

### 3.1. Prevalence of Valve Involvement

Echocardiography was performed at a median of three days after admission (IQR 2–5 days; median length of stay 7 days), by which time decongestive therapy had been under way for several days. Among the 530 patients, 378 (71.3%) had at least one significantly diseased valve. The mitral valve was most frequently involved (235 patients, 44.3%), followed closely by the tricuspid valve (232, 43.8%) and then the aortic valve (146, 27.5%). At the level of individual lesions, moderate or severe tricuspid regurgitation (43.8%) and mitral regurgitation (42.8%) predominated, with aortic stenosis present in 19.8%, aortic regurgitation in 12.6% and mitral stenosis in 4.2% (Table 1). Multivalvular disease, defined as involvement of two or more valves, was present in 185 patients (34.9%).

### 3.2. Clinical Phenotype of Multivalvular Involvement

Patients with multivalvular disease differed substantially from those with single or no valve involvement (Table 2). They were older (median 83 vs. 79 years, *p* < 0.001), far more often had acute-on-chronic rather than de novo heart failure (de novo in 16.2% vs. 33.6%, *p* < 0.001), more frequently had AF (67.0% vs. 54.8%, *p* = 0.008), and were markedly frailer (CFS ≥ 5 in 83.8% vs. 57.1%, *p* < 0.001). They were more symptomatic at baseline, with NYHA class IV in 27.0% versus 9.9% (*p* < 0.001), and carried a heavier burden of CKD (61.6% vs. 46.4%, *p* = 0.001), with higher baseline creatinine (1.20 vs. 1.10 mg/dL, *p* = 0.008), lower baseline eGFR (54 vs. 64 mL/min/1.73 m^2^, *p* < 0.001), a shift towards more advanced KDIGO stages (*p* = 0.002) and higher NT-proBNP (median 7674 vs. 4520 pg/mL, *p* < 0.001), while several other comorbidities—hypertension, prior stroke, malignancy and prior device therapy—did not differ materially between groups. Coronary status differed across the three categories recorded (*p* = 0.005), but this reflected ascertainment rather than disease: patients with multivalvular involvement were less likely to have undergone coronary assessment (not assessed in 42.2% versus 28.4%), and among those in whom the coronary anatomy had been evaluated the prevalence of documented disease was almost identical (69.2% versus 66.4%, *p* = 0.70). Diabetes was somewhat less frequent in the multivalvular group (42.7% vs. 56.8%, *p* = 0.003).

On echocardiographic assessment, multivalvular involvement was associated with higher E/e’ ratio and more advanced remodeling: left atrial volume index (LAVi) was higher (59.5 vs. 48.5 mL/m^2^, *p* < 0.001), RV function was worse (TAPSE 17.0 vs. 18.0 mm and RV S′ 9.5 vs. 11.0 cm/s, both *p* < 0.001; RV dilation 35.7% vs. 18.6%, *p* < 0.001), and estimated PASP was higher (50 vs. 40 mmHg, *p* < 0.001), with concomitant right HF roughly twice as frequent (60.5% vs. 33.0%, *p* < 0.001). LVEF did not differ between groups (*p* = 0.38), and the distribution of EF categories was also similar (LVEF < 35% in 31.4% vs. 31.3%, *p* = 0.35), arguing against a major imbalance in severe left ventricular systolic dysfunction as an explanation for the observed outcome difference. AF, when present, was more often persistent or permanent in the multivalvular group (47.6% vs. 27.8%; *p* = 0.002 across patterns), consistent with a more advanced atrial remodeling phenotype. Medical therapy also differed in a clinically relevant direction (Table 3): at discharge, patients with multivalvular involvement less often received renin–angiotensin system inhibition (angiotensin-converting enzyme [ACE] inhibitor or angiotensin receptor blocker [ARB] 30.4% vs. 43.6%, *p* = 0.006; angiotensin receptor–neprilysin inhibitor [ARNI] 7.1% vs. 12.8%, *p* = 0.075) and sodium–glucose cotransporter-2 (SGLT2) inhibitors (58.3% vs. 67.2%, *p* = 0.064), received higher furosemide doses, and, among those with LVEF < 40%, were prescribed fewer disease-modifying pillars (*p* < 0.001).

### 3.3. Patterns of Multivalvular Involvement

Among the 185 patients with multivalvular disease, involvement was not distributed at random across the three valves (Table 4). Combined mitral and tricuspid disease was by far the most frequent pattern, accounting for 89 patients (48.1% of all multivalvular cases), followed by aortic–mitral involvement (30 patients, 16.2%) and aortic–tricuspid involvement (16 patients, 8.6%); the remaining 50 patients (27.0%) had all three valves involved. At the level of individual lesions, the combination of moderate-or-severe mitral and tricuspid regurgitation dominated, being present in 133 of the 185 multivalvular patients (71.9%), followed by mitral regurgitation with aortic regurgitation (25.4%) and mitral regurgitation with aortic stenosis (24.9%).

One-year mortality also varied by pattern: it was 38.2% with mitral–tricuspid involvement, 33.3% with aortic–mitral and 31.2% with aortic–tricuspid involvement, and highest at 54.0% in those with all three valves involved. These pattern-specific estimates are descriptive, derived from small subgroups, and were not intended for formal between-pattern comparison.

### 3.4. Valve Burden and One-Year Mortality

**Figure 1:**
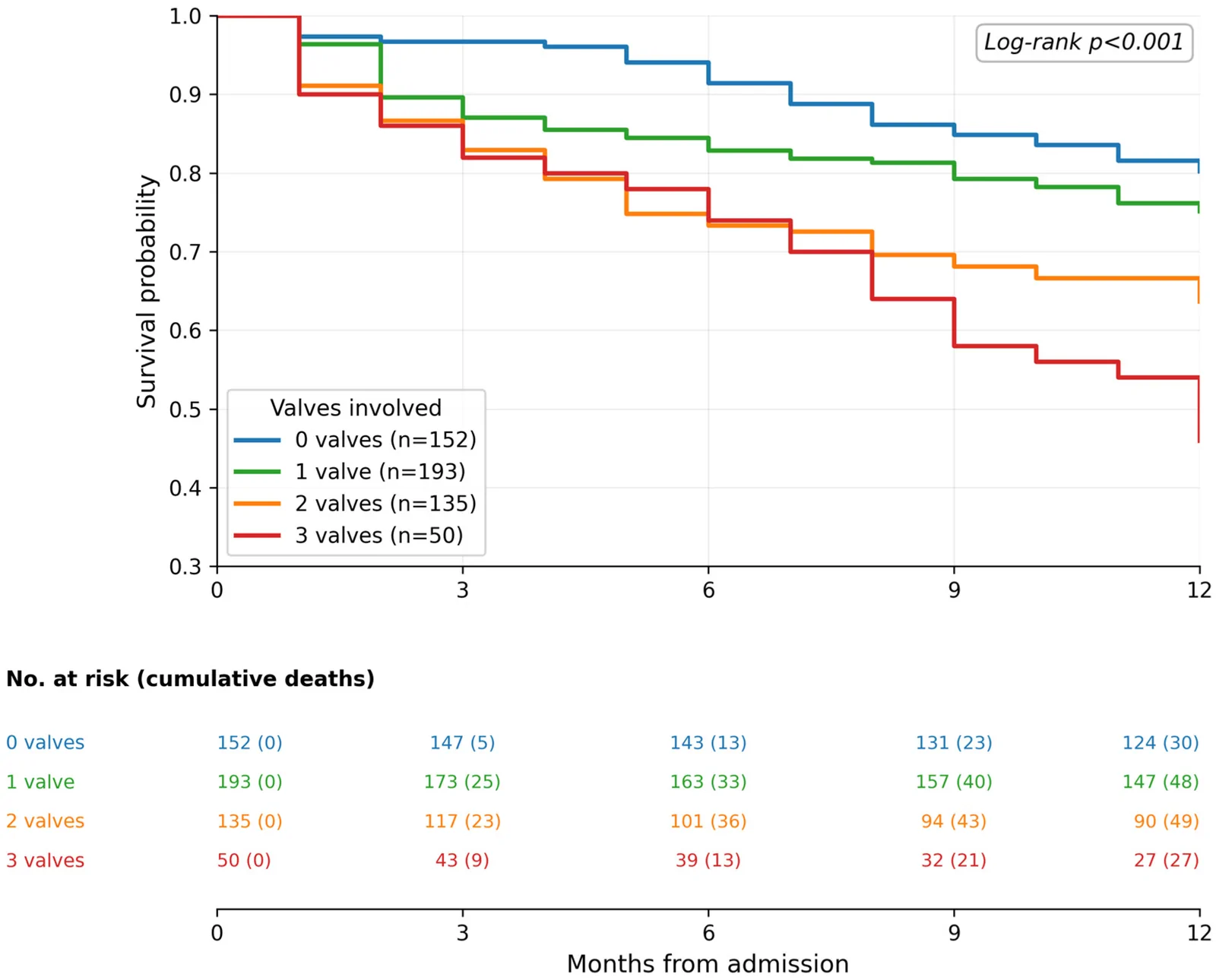
Kaplan–Meier survival by number of involved valves (0–3) over 12 months. Survival diverges progressively with increasing valve burden (log-rank *p* < 0.001). Numbers below the plot are patients at risk with cumulative deaths in parentheses. These curves are unadjusted and do not account for the substantial baseline differences between valve-burden groups; model-based adjusted survival curves are shown in Supplementary Figure S3.

One-year all-cause mortality in the whole cohort was 29.1% (154 deaths). Mortality rose stepwise with the number of involved valves: 19.7% with no valve involvement, 24.9% with one, 36.3% with two and 54.0% with three valves (log-rank *p* < 0.001; Table 5, Figure 1). Modeled continuously, each additional involved valve was associated with a crude HR for death of 1.51 (95% CI 1.28–1.78, *p* < 0.001). Multivalvular disease (≥2 valves) versus single or no involvement carried a crude HR of 2.07 (1.51–2.84, *p* < 0.001).

The association persisted after multivariable adjustment. The four adjustment covariates were specified a priori on clinical grounds and constrained in number to preserve the events-per-variable ratio (Section 2.5); right-sided variables were deliberately excluded from the primary model and are examined separately in Section 3.6. Because 11 patients had a missing admission NT-proBNP, the complete-case adjusted models throughout include 519 patients, with 149, 159 and 253 events for mortality, the composite of death or RRT and the triple composite respectively; the corresponding full-cohort event counts are 154, 164 and 258. In the Cox model (n = 519, 149 events) incorporating admission log NT-proBNP, frailty and age, each additional involved valve remained associated with mortality (adjusted HR 1.22, 95% CI 1.03–1.45, *p* = 0.023), as did multivalvular disease as a dichotomy (adjusted HR 1.48, 95% CI 1.06–2.07, *p* = 0.020) (Table 6; full model output in Supplementary Table S2). The proportional-hazards assumption was satisfied for every term (all *p* > 0.12). Modeling valve count categorically gave adjusted HRs relative to no valve involvement of 1.02 (0.64–1.63) for one valve, 1.41 (0.88–2.27) for two and 1.69 (0.98–2.91) for three; a likelihood-ratio test comparing this parameterization with the continuous term gave no evidence of departure from linearity (*p* = 0.71) (Supplementary Table S3). No individual category reached significance against the reference, so the continuous term should be read as an average gradient rather than as evidence of a discrete threshold, and the adjusted gradient was driven predominantly by patients with two or three involved valves. The attenuation of the HRs on adjustment indicates that a substantial part of the prognostic signal of valve burden is carried by the more advanced congestive and frail phenotype of these patients.

### 3.5. Renal Function, Cardiorenal and Composite Outcomes

Valve burden tracked closely with chronic renal impairment (Table 7). Baseline eGFR declined monotonically across strata, from a median of 66 mL/min/1.73 m^2^ in patients with no valve involvement to 47 mL/min/1.73 m^2^ in those with three valves involved (*p* < 0.001), and the proportion with KDIGO stage 3 or worse CKD rose in parallel from 43.4% to 68% (*p* = 0.004). To test whether multivalvular disease also precipitates acute cardiorenal injury during decompensation, in-hospital AKI was examined across the same strata; it was not, occurring in 45.4%, 48.2%, 50.4% and 50.0% of patients (*p* = 0.85). Valve burden therefore tracked chronic renal impairment but not acute in-hospital renal deterioration.

Both composite outcomes showed steep, monotonic gradients. The cardiorenal composite of death or RRT occurred in 21.1%, 25.9%, 38.5% and 60.0% of patients across increasing valve burden, and the triple composite of death, AHF rehospitalization or RRT in 38.2%, 44.6%, 54.8% and 80.0% (both *p* < 0.001). After adjustment for admission log NT-proBNP, frailty and age, each additional involved valve was associated with an HR of 1.26 (95% CI 1.06–1.48, *p* = 0.007) for death or RRT and 1.21 (1.06–1.39, *p* = 0.004) for the triple composite; the corresponding adjusted estimates for multivalvular disease as a dichotomy were 1.55 (1.13–2.14, *p* = 0.007) and 1.39 (1.08–1.80, *p* = 0.011) (Table 7; full model output in Supplementary Table S4, and Kaplan–Meier curves for the cardiorenal composite in Supplementary Figure S1). A similar association was observed for the cardiorenal composite; however this endpoint added only 10 events beyond the 154 deaths and was therefore driven predominantly by mortality, precluding interpretation as evidence of a distinct renal prognostic association.

The relationship was also apparent in the reverse direction: the prevalence of multivalvular disease rose from 16.7% in KDIGO stage 1 to 52.5% in stage 4 (*p* = 0.002), and baseline eGFR correlated inversely with valve count (Spearman ρ = −0.18, *p* < 0.001), with tricuspid involvement showing the steepest gradient (Supplementary Table S5). This cross-sectional association was substantially attenuated after adjustment for age, sex and AF (odds ratio 1.45, 95% CI 0.98–2.12, *p* = 0.06), indicating that much of it reflects determinants common to both conditions rather than a direct effect of either upon the other.

To establish whether these associations simply reflected pre-existing renal impairment, baseline eGFR was added to the multivariable models. The estimates were essentially unchanged: for one-year mortality the adjusted HR per additional involved valve was 1.22 (1.03–1.45) both with and without baseline eGFR in the model, and for death or RRT 1.26 (1.06–1.48) both with and without, with the corresponding estimate for multivalvular disease as a dichotomy unchanged at 1.55. Nor was there evidence that the association differed according to established CKD (interaction *p* = 0.31 for mortality and *p* = 0.19 for the cardiorenal composite): the gradient for death or RRT was apparent in patients without CKD (adjusted HR 1.37 per additional valve, 95% CI 1.02–1.85) and of similar direction in those with KDIGO stage 3 or worse (1.20, 0.98–1.47). Adjustment for baseline eGFR did not materially alter the estimated association between valve burden and the outcomes examined.

### 3.6. Sensitivity Analyses and Model Checks

Because definitions of multivalvular disease differ between studies, the primary analysis was repeated using three progressively stricter alternatives (Table 8). Applying a severe-lesion threshold to two or more valves identified only 39 patients (7.4%), among whom one-year mortality was 48.7% versus 27.5% (crude HR 2.01, 95% CI 1.25–3.25); the adjusted estimate was directionally similar but no longer reached significance (HR 1.43, 95% CI 0.88–2.33, *p* = 0.15), consistent with the small number of patients meeting this definition. Requiring at least one severe lesion together with at least moderate disease on a second valve identified 116 patients (21.9%) and yielded an adjusted HR of 1.60 (1.14–2.25, *p* = 0.007). A left-sided definition analogous to that used in European surveys—severe aortic or mitral disease together with at least moderate disease of the other left-sided valve—identified 44 patients (8.3%), in whom one-year mortality reached 59.1% and the adjusted association was the strongest of all (HR 1.85, 95% CI 1.20–2.86, *p* = 0.005). Across all four definitions the direction and approximate magnitude of the association were preserved.

Replacing the simple count with a severity-weighted score summing the grade of each valve yielded a closely comparable result (adjusted HR 1.15 per point, 95% CI 1.04–1.28, *p* = 0.009) and near-identical discrimination (Harrell’s C 0.715 versus 0.714). Weighting by severity therefore added nothing to simple enumeration, and the count was retained as the more parsimonious and more readily applied measure (Supplementary Table S6).

When the three valves were entered simultaneously in an adjusted model, no single valve reached significance: the adjusted HRs were 1.35 (0.95–1.91, *p* = 0.09) for mitral, 1.12 (0.80–1.59, *p* = 0.51) for aortic and 1.16 (0.83–1.64, *p* = 0.38) for tricuspid involvement. The prognostic signal thus appears to reside in the accumulation of lesions rather than in any dominant individual valve. There was no evidence that the association differed by LVEF phenotype (interaction *p* = 0.30), with adjusted HRs per additional valve of 1.27 (1.01–1.61) in patients with LVEF < 50% and 1.15 (0.89–1.50) in those with LVEF ≥ 50%.

The proportional-hazards assumption, examined using scaled Schoenfeld residuals, was satisfied for the exposure (*p* = 0.84) and for every covariate in the primary model (all *p* > 0.12).

Finally, an expanded model additionally adjusting for sex, AF, LVEF, baseline eGFR and de novo versus acute-on-chronic status did not materially alter the estimates: the adjusted HR was 1.19 (1.01–1.42, *p* = 0.043) per additional valve and 1.44 (1.03–2.01, *p* = 0.033) for multivalvular disease, with a modest improvement in discrimination (Harrell’s C 0.728 versus 0.714) (Supplementary Table S7). Of the added covariates only de novo presentation carried independent prognostic weight (HR 0.49, 0.29–0.82, *p* = 0.007); LVEF, in particular, was not independently associated with mortality (HR 1.01 per %, 0.99–1.02, *p* = 0.42), nor were sex, AF or baseline eGFR. Because right-sided involvement is closely bound up with tricuspid disease, a further set of sensitivity models added right-sided variables individually to the primary model (Supplementary Table S8). Every such adjustment attenuated the estimate to non-significance: 1.18 (0.98–1.42, *p* = 0.083) with PASP, 1.18 (0.99–1.41, *p* = 0.058) with TAPSE, 1.19 (1.00–1.42, *p* = 0.051) with tricuspid annular S′, 1.18 (0.99–1.41, *p* = 0.065) with RV dilation, and 1.15 (0.96–1.37, *p* = 0.130) with clinically defined right HF; entering all four together gave 1.13 (0.94–1.37, *p* = 0.195). The corresponding estimates for the dichotomy were 1.33 (0.94–1.87, *p* = 0.103) and 1.29 (0.91–1.85, *p* = 0.154). Stratified analysis was concordant: among the 226 patients with clinically manifest right HF the crude gradient was steep (crude HR 1.52 per valve, 1.22–1.90, *p* < 0.001; adjusted 1.22, 0.97–1.55, *p* = 0.09), whereas among the 304 patients without it no association was detectable (crude 1.25, 0.96–1.63, *p* = 0.10; adjusted 1.06, 0.80–1.41, *p* = 0.69). One-year mortality across zero to three involved valves was 20.7%, 19.2%, 21.4% and 47.1% in the absence of right HF but 17.1%, 34.2%, 46.8% and 57.6% in its presence, while the prevalence of right HF itself rose from 27.0% to 66.0% across the same strata. The formal interaction was not significant (*p* = 0.45) and the subgroup without right HF contained only 66 deaths, so effect modification cannot be claimed (Supplementary Table S9); the consistent pattern nonetheless indicates that the prognostic information carried by valve burden is closely bound up with right-sided involvement. Adjustment for clinically manifest right HF yielded similar point estimates for mortality (1.15, 0.96–1.37, *p* = 0.130), death or RRT (1.19, 1.00–1.41, *p* = 0.051) and the triple composite (1.19, 1.03–1.36, *p* = 0.015), although statistical significance was retained only for the triple composite. Because these endpoints differ in composition, the greater precision of the composite analyses cannot determine whether the non-significant mortality association reflects limited power or true attenuation. Stratification was concordant for both mortality and the triple composite, with no association detectable in the absence of right HF. Because severe aortic stenosis became more frequent with increasing valve burden (0%, 14.5%, 11.9% and 42.0% across strata; *p* < 0.001) (Supplementary Table S10), it was added to the primary model: the estimate fell to 1.18 per additional valve (0.99–1.41, *p* = 0.060) and 1.44 for the dichotomy (1.03–2.01, *p* = 0.034) (Supplementary Table S11). Adding the number of disease-modifying agents taken at admission left the estimates unchanged. Finally, because the groups differed across many baseline characteristics, a propensity score for multivalvular involvement was constructed from age, sex, AF, LVEF, baseline eGFR, de novo status, frailty, log NT-proBNP, CKD and right HF (C-statistic 0.738). Neither propensity-score adjustment (HR 1.33, 0.94–1.87, *p* = 0.10) nor stabilized inverse-probability weighting (1.31, 0.92–1.86, *p* = 0.13) reproduced a significant association with mortality, and the estimate per additional valve was 1.15 (0.96–1.37, *p* = 0.13). Because right HF may act as an intermediate rather than a conventional confounder, the propensity model was refitted without it: the association was then retained (propensity-score-adjusted HR 1.43, 1.02–2.00, *p* = 0.036; 1.19 per additional valve, 1.00–1.41, *p* = 0.050; inverse-probability weighted 1.40, 0.99–1.98, *p* = 0.055). The propensity analyses therefore reproduce, rather than independently corroborate, the pattern seen with direct adjustment: the estimate is retained when only baseline characteristics are balanced and lost when right-sided involvement is included. Finally, because the index hospitalization and the post-discharge period may represent different prognostic processes, the primary model was repeated as a post-discharge sensitivity analysis among the 503 hospital survivors with time zero at discharge (n = 494 with complete covariates, 124 deaths). The crude gradient was preserved (HR 1.45 per additional valve, 1.21–1.73, *p* < 0.001; 1.89 for the dichotomy, 1.33–2.68, *p* < 0.001), whereas the adjusted associations were attenuated and did not reach statistical significance (1.17, 0.97–1.41, *p* = 0.094 per valve; 1.35, 0.94–1.94, *p* = 0.110 for the dichotomy) (Supplementary Table S12).

## 4. Discussion

### 4.1. Principal Findings

In a prospective cohort of 530 consecutive patients hospitalized for AHF, significant valvular involvement was the rule rather than the exception, and its cumulative extent tracked closely with outcome. Six findings stand out. First, almost three quarters of patients had at least one significantly diseased valve and more than a third had two or more, a burden appreciably higher than that reported in referral-based valve surveys. Second, involvement was far from randomly distributed: the mitral–tricuspid combination dominated, accounting for close to half of all multivalvular cases, with all three valves involved in a further quarter. Third, multivalvular disease identified an older, frailer, more congested and more renally impaired phenotype, characterized hemodynamically by elevated filling pressures, atrial enlargement and right-sided involvement rather than by lower EF. Fourth, valve burden tracked closely with chronic renal impairment but not with acute in-hospital renal deterioration, and its association was preserved for the cardiorenal composite of death or RRT. Fifth, one-year mortality rose stepwise with each additional affected valve; this gradient persisted, although attenuated, after adjustment for natriuretic peptides, frailty and age, and its direction was reproduced across three progressively stricter definitions of multivalvular disease. Sixth, and central to interpretation, the adjusted association with mortality did not survive adjustment for any measure of right-sided involvement, was not reproduced in propensity-based analyses that included right HF, whereas it was retained when right HF was omitted from the propensity model, and was not detectable, for either mortality or the triple composite, in patients without clinically manifest right HF. Taken together these analyses do not support an independent prognostic role of valve burden for mortality, and indicate that the information it conveys is largely that of the accompanying right-heart phenotype.

### 4.2. Prevalence in the Context of Existing Series

Our prevalence estimates lie at the upper end of the published range, and the reasons are instructive rather than incidental. In the ESC EURObservational Research Programme (ESC-EORP) Valvular Heart Disease II survey, which enrolled patients referred with severe native valve disease, multiple left-sided involvement accounted for approximately one quarter of the cohort [14,15], and contemporary echocardiographic series restricted to severe lesions report multivalvular disease in a smaller minority still. Our figure of 34.9% exceeds these, and three differences account for the discrepancy: ascertainment, since our cohort was defined by an AHF admission rather than by referral for valve disease, and valvular involvement is recognized to be highly prevalent in this setting [2]; threshold, since we counted lesions of at least moderate severity whereas the comparator series required severe disease; and age, since with a median of 83 years in the multivalvular group our population is older than most valve registries and both degenerative and functional lesions accumulate with advancing age [3,9].

Our prevalence figures should therefore not be read as directly comparable with severity-restricted surveys. They do, however, describe the population in which the question is arguably most pressing—the elderly, multimorbid patient presenting acutely, in whom several moderate lesions coexist and no single one dominates the clinical picture. It is precisely this configuration that current recommendations, organized around isolated severe lesions, address least well [4,9].

### 4.3. Patterns of Involvement: A Heart-Failure Signature

The distribution of valve combinations in our cohort was distinctly non-random and characteristic of the AHF setting. Mitral–tricuspid involvement was the dominant pattern, followed by aortic–mitral and then aortic–tricuspid, and at the lesion level the pairing of moderate-or-severe mitral with tricuspid regurgitation was present in close to three quarters of multivalvular patients. This ordering is consistent with the substantial burden of combined mitral and tricuspid regurgitation described in HF registries [2,16], but differs from that observed in surveys of predominantly severe, referral-based valve disease, in which left-sided combinations such as aortic stenosis with mitral regurgitation predominate [15].

The divergence is readily explained mechanistically. Left-sided volume and pressure overload with progressive atrial and annular dilation—very often in the setting of AF, present in two thirds of our multivalvular patients—generates functional mitral and tricuspid regurgitation in tandem, the tricuspid lesion being further promoted by pulmonary hypertension and RV and atrial dilation [5,9]. Each lesion is independently associated with adverse outcome in community populations [17,18], and their frequent coexistence here reflects a shared upstream substrate rather than two unrelated processes, whereas aortic disease follows a largely independent calcific trajectory. The predominance of the mitral–tricuspid pattern therefore suggests that AHF multivalvular involvement is, to a considerable degree, a consequence of advanced cardiac remodeling rather than an expression of primary valve pathology—a reading supported by the phenotype we observed.

### 4.4. Multivalvular Burden as a Marker of Advanced Remodeling and of Prognosis

Patients with multivalvular involvement were older, markedly frailer, more often in AF, more congested and of lower renal reserve, with higher NT-proBNP and lower eGFR. Echocardiography demonstrated higher E/e′ ratios, greater left atrial enlargement, impaired RV function, and elevated pulmonary pressures, accompanied by an approximately twofold higher prevalence of right HF— yet LVEF did not differ. This constellation is that of a remodeled left atrium and a strained right ventricle, not simply a more impaired left ventricle. It parallels the multisystem, low-reserve profile documented in contemporary AHF cohorts [2], and indicates that valve burden and systolic dysfunction index different aspects of disease. The lower prevalence of diabetes in the multivalvular group most plausibly reflects the older, more degenerative substrate of this phenotype rather than any protective effect. That prior valve replacement was equally distributed between groups argues against the valve burden being an artifact of previously intervened disease.

Against that background, our central observation is that the number of significantly diseased valves behaves as a graded prognostic marker: one-year mortality rose from approximately one fifth of patients with no valve involvement to more than half of those with all three valves affected. Whereas most prognostic work in valvular heart disease considers lesions individually, this stepwise gradient suggests that cumulative burden integrates information about the severity, chronicity and multisystem consequences of the underlying cardiac disease that no single lesion conveys.

The partial attenuation of the HR on adjustment—from 1.51 to 1.22 per additional valve—is itself informative. A substantial part of the prognostic signal of valve burden is carried by the congestive and frail phenotype that accompanies it, in which renal dysfunction [19] and frailty [20] independently drive outcome in AHF; multivalvular disease is therefore, in considerable measure, a marker of that phenotype. An incremental association persisted after the baseline adjustment set and after expanded adjustment, but it did not persist once right-sided involvement was accounted for. This is biologically plausible: coexisting lesions interact hemodynamically, and their combined effect on ventricular loading, forward flow and filling pressures may exceed that of either lesion considered alone—interactions examined in detail for the combination of aortic stenosis and mitral regurgitation [5,6,21]. Equally, one lesion may mask another, so that the aggregate hemodynamic penalty is underestimated when each is graded in isolation [9]. Valve burden thus appears to function primarily as an integrative marker of advanced remodeling, congestion and reduced physiological reserve rather than as an independent determinant of outcome. The pattern of attenuation is informative in its own right: every adjustment for right-sided involvement—pulmonary pressure, RV function, RV dilation or clinically manifest right HF—removed statistical significance for mortality, though not for the triple composite endpoint, which differs in composition and accrued more events. The more robust observation is that the gradient across valve strata was confined, for both mortality and the triple composite, to patients whose right heart was already clinically involved. Propensity-based analyses yielded similarly non-significant estimates; however, because clinically manifest right HF was included in the propensity model, these analyses should also be interpreted as conditional on the measured right-heart phenotype. When the propensity model was refitted without right HF the association was retained, which supports the interpretation that the attenuation is principally associated with conditioning on the right-sided variables rather than in baseline imbalance more generally. A directed acyclic graph summarizing the assumed relationships between valve burden, right-sided involvement, the shared substrate and mortality is provided in Supplementary Figure S2. Right HF is a downstream consequence of significant tricuspid regurgitation, so this is the behavior expected of an intermediate on the pathway from valve involvement to death rather than of a competing predictor. This distinction matters for interpretation. Adjustment for a confounder removes a spurious component of an association, whereas adjustment for a variable that lies on the causal pathway removes the very mechanism through which the exposure acts, and will attenuate or abolish the estimate however real the underlying effect. The loss of significance after conditioning on right-sided involvement is therefore the behavior expected of mediator adjustment and should not be read as evidence that the study design has failed to detect a prognostic signal. Because these right-sided variables are plausibly intermediates rather than confounders, their inclusion cannot establish that valve burden lacks independent prognostic weight; what the pattern does show is that much of the prognostic information conveyed by cumulative valve burden is closely linked to the accompanying right-heart phenotype, and that accumulating valve involvement marks the point at which the right heart has become clinically prominent.

Our adjusted estimates are smaller than those reported in a recent analysis of moderate aortic regurgitation, in which concomitant moderate valve disease carried a hazard comparable to that of isolated significant aortic regurgitation [7]; that study examined an ambulatory population with preserved EF, whereas our cohort comprises acutely decompensated, elderly and frequently frail patients in whom competing non-valvular determinants of mortality are far more prominent.

### 4.5. A Cardiorenal Dimension: Chronic Impairment Without Acute Injury

The renal findings are internally dissociated. Valve burden was strongly and monotonically related to chronic renal impairment: baseline eGFR fell by almost twenty milliliters per minute across the range of valve involvement, and the prevalence of stage 3 or worse CKD rose from just over two fifths to more than two thirds. Yet in-hospital AKI was entirely flat across the same strata. Had that been the dominant mechanism, an excess of in-hospital AKI would have been expected, and it was not observed. The pattern instead suggests a shared chronic substrate: advancing age, long-standing pressure and volume overload, systemic venous congestion and the accumulated vascular and degenerative burden that produces multivalvular involvement also erode renal reserve over years, so that the two evolve in parallel rather than one acutely causing the other. This is consistent with the contemporary understanding of cardiorenal syndrome as a spectrum of bidirectional heart–kidney interactions rather than a single mechanism [22,23].

Examined in the reverse direction, patients with more advanced baseline CKD carried a heavier valvular burden, the prevalence of multivalvular disease rising from roughly one in six in KDIGO stage 1 to more than half in stage 4. That this cross-sectional gradient largely dissolved on adjustment for age, sex and AF indicates that the two conditions accumulate together because they share determinants rather than because either directly generates the other; the direction of causation cannot be resolved in data of this kind [24]. The association mirrors a relationship well described outside the acute setting [25,26,27,28], and our data extend it to the acute setting while indicating that it is not confined to calcific left-sided lesions, tricuspid involvement showing the steepest gradient across renal stages. Most importantly, adding baseline eGFR to the models left the estimates for valve burden essentially unchanged, and the association was equally evident in patients with and without established CKD; valve burden therefore does not act merely as a surrogate for pre-existing renal impairment.

The association of valve burden with the composite of death or RRT was of similar direction and magnitude to that observed for mortality; that composite added only 10 events beyond the 154 deaths, however, and is therefore driven predominantly by mortality rather than indicating a distinct renal prognostic association. We would not read more into the composite than this: it added few events beyond death (159 versus 149 in adjusted models), the CIs overlap substantially, and no formal comparison of the HRs was undertaken. Isolated RRT was not modeled separately, since the few renal events and the competing risk of death would make a cause-specific analysis imprecise.

### 4.6. The Severity Threshold and the Robustness of the Association

Definitions of multivalvular disease are not standardized, and the choice of severity threshold is therefore a substantive determinant of what is being measured. Some series restrict the term to left-sided disease, requiring a severe native lesion on one left-sided valve together with at least moderate disease on the other ipsilateral valve [14], whereas in HF populations the tricuspid valve is frequently involved and is increasingly regarded as an integral component of overall valvular burden [2]. We deliberately adopted a more inclusive threshold for two reasons. First, in AHF functional mitral and tricuspid regurgitation predominate and carry prognostic weight even at moderate grade, so a criterion restricted to severe lesions would exclude much of the valve burden actually encountered at the bedside; the recent finding that a combination of moderate lesions carries a risk comparable to that of an isolated significant lesion supports this choice [7,8]. Second, our aim was to test whether the simple accumulation of hemodynamically significant lesions conveys prognostic information, rather than to characterize advanced surgical valve disease.

The sensitivity analyses were designed to establish whether this choice determined the result. Restricting the definition to severe lesions identified only 39 patients (7.4%), among whom the crude association remained significant but the adjusted estimate lost statistical significance—an outcome readily attributable to the small number of patients meeting this definition rather than to absence of effect. Broadening the definition to require one severe and one at least moderate lesion, or applying a left-sided definition analogous to European surveys, yielded adjusted HRs of 1.60 and 1.85 respectively. Across all four definitions the direction of the association was preserved and its magnitude broadly comparable, indicating that our findings are not an artifact of a permissive threshold; if anything, the stricter left-sided definition identified the highest-risk subgroup, with one-year mortality approaching 60%. The corollary is that lesions of moderate severity, when they coexist, are not benign—consonant with the emerging view that risk in valvular heart disease is continuous and that a framework built on isolated severe lesions may fail to capture cumulative hemodynamic burden [7,8]. We would nonetheless echo the caution that accompanies that literature: observational data of this kind establish prognostic relevance, not intervention thresholds [8,9].

### 4.7. Clinical Implications

Valve burden should not be regarded as an independently validated prognostic factor for mortality, and the present data do not support its use as a risk marker in its own right. What it offers is descriptive: the practical appeal of enumerating involved valves lies in its simplicity. The number of significantly diseased valves is available from the same routine transthoracic study already performed in every AHF admission and requires no additional imaging, computation or laboratory input. Valve count should not, however, replace lesion-specific assessment or established prognostic markers, and it is proposed here as a descriptive marker of an adverse phenotype rather than as a validated or independent risk score. Its practical value lies in what it points to: a patient with two or three significantly involved valves should prompt deliberate assessment of the right heart, because the observed association was closely linked to right-sided involvement and association with outcome was substantially attenuated after accounting for the accompanying phenotype, and in particular for right-sided involvement, so it should be interpreted alongside frailty, renal function, natriuretic peptides and right-heart assessment rather than in isolation, and the adjusted gradient in our data was driven predominantly by patients with two or three involved valves rather than by the step from none to one. Dedicated recommendations for these patients remain scarce, with existing guidance resting largely on small retrospective series and expert consensus, and with such patients underrepresented in randomized trials [4,9]. Pending the results of the prospective multivalvular registries now underway [9], simple descriptors of cumulative burden may serve as a useful interim aid for identifying patients who warrant comprehensive evaluation and closer follow-up.

### 4.8. Limitations

Several limitations should be acknowledged. This was a single-center observational study of an elderly Mediterranean cohort, and residual confounding cannot be excluded despite adjustment for major prognostic covariates; because several covariates are plausibly mediators as well as confounders, the adjusted estimates should be read as the association remaining after accounting for the accompanying clinical phenotype rather than as the total prognostic association of valve burden. No external validation was performed.

Echocardiographic examinations were interpreted by two separate experienced cardiologists as part of routine clinical care; studies were not re-adjudicated by an independent core laboratory, and formal interobserver reproducibility was not assessed. Misclassification of lesion severity, particularly for loading-dependent mitral and tricuspid regurgitation, therefore cannot be excluded. Studies were performed at a median of three days after admission, once decongestion was generally under way, reducing overestimation of functional regurgitation; however, timing was not standardized to a euvolemic state, and the mechanism of individual mitral and tricuspid lesions was not systematically recorded. Echocardiography reflected a single assessment during the index hospitalization, so the trajectory of valve disease over time was not captured. Several conventional echocardiographic parameters were validated in single-valve disease and may be less reliable when lesions coexist [9]. The adjusted association of valve burden with mortality did not survive adjustment for any measure of right-sided involvement, and was not detectable among patients without clinically manifest right HF. Because right-sided involvement plausibly lies on the causal pathway from tricuspid disease to death, this cannot be read as evidence against a contributory role of valve burden; it does mean, however, that valve burden and right-heart involvement cannot be prognostically separated in this cohort, and that the mortality analysis cannot support a claim of independent prognostic value. The triple composite endpoint retained an association after right-sided adjustment, but because it differs in composition from mortality this cannot be attributed to statistical power alone. The interaction test was not significant (*p* = 0.45) and the subgroup without right HF contained only 66 deaths, so the stratified comparison is underpowered and hypothesis-generating. The adjusted association was likewise attenuated and no longer significant in a post-discharge sensitivity analysis starting at discharge, in which the 27 in-hospital deaths are excluded by design. The estimates remained directionally consistent, but this analysis cannot distinguish reduced power from a contribution of the in-hospital period to the overall association.

Valve involvement was defined by a threshold of at least moderate severity applied at the valve level; this definition is deliberately more inclusive than those requiring severe lesions or restricted to left-sided disease, so our prevalence figures are not directly comparable with series confined to severe valvular heart disease, although the sensitivity analyses yielded consistent associations. Mixed single-valve disease was counted as involvement of one valve, and the pulmonary valve was not systematically quantified. A severity-weighted score performed no better than the simple count, but neither captures the hemodynamic interaction between specific lesion combinations. In patients with a previously implanted prosthesis, a normally functioning prosthetic valve was not counted as valve involvement; while this convention correctly captures hemodynamically significant disease at the index admission, it may underestimate the cumulative structural consequences of the antecedent native lesion.

The number of patients with three-valve involvement, and with severe disease of two or more valves, was modest, limiting precision at the extremes of burden, and RRT events were few, so the cardiorenal composite is driven predominantly by death. Non-fatal events were ascertained at scheduled checkpoints, so although the order of recorded composite events was reconstructed from the available event-time variables, rehospitalizations not entered in either the binary follow-up fields or the composite event-time variables could not be recovered. The direction of any resulting bias cannot be determined with confidence, since the likelihood of an unrecorded rehospitalization may itself be related to valve burden. Baseline creatinine was selected clinically as the most representative stable pre-admission value rather than by an automated rule, which may introduce measurement error into baseline eGFR and KDIGO staging. The mechanism of individual mitral and tricuspid lesions (primary versus secondary) was not recorded, so the dominant mitral–tricuspid pattern cannot be formally attributed to functional regurgitation, although the excess of persistent or permanent AF and of atrial enlargement is consistent with that interpretation. Severe aortic stenosis was not phenotyped hemodynamically, since transvalvular gradients, valve area and stroke volume index were not captured in the registry; low-flow, low-gradient aortic stenosis could therefore not be identified, and adjustment for severe aortic stenosis as a binary variable attenuated the mortality estimate to borderline significance. No patient had cardiac resynchronization therapy at baseline, and new device implantation during follow-up was not recorded. Medical therapy at discharge differed between groups, with less renin–angiotensin system inhibition and fewer disease-modifying pillars in the multivalvular group [29,30]; although adjustment for admission therapy did not alter the estimates, differences in treatment implementation remain a plausible source of residual confounding. Valve interventions and changes in medical therapy during the follow-up period were not recorded, so their potential to modify the relationship between valve burden and outcome could not be assessed; this is relevant because patients with greater valve burden may be more likely to be referred for intervention. Subgroup and sensitivity analyses were not corrected for multiplicity and should be regarded as exploratory. Finally, cause of post-discharge death could not be reliably adjudicated; the primary endpoint was therefore analyzed as all-cause mortality.

## 5. Conclusions

In patients hospitalized with AHF, multivalvular involvement was common, followed a predominantly mitral–tricuspid pattern, and identified an older, frailer, more advanced HF phenotype with substantial right-sided and chronic renal involvement. One-year mortality increased progressively with the number of significantly involved valves, and this association persisted after adjustment for admission natriuretic peptides, frailty and age. After adjustment for right-sided involvement, including propensity-based adjustment incorporating clinically manifest right HF, the association was no longer statistically significant for mortality, although it persisted for the triple composite endpoint and it was not detectable for either mortality or the triple composite endpoint in patients without clinically manifest right HF. The attenuation of the association after adjustment for right-sided variables is what adjustment for an intermediate is expected to produce and suggests that much of the prognostic information conveyed by cumulative valve burden is closely linked to the accompanying right-heart phenotype. Cumulative valve burden is therefore best interpreted as a powerful phenotypic marker of that phenotype rather than as an independent causal determinant of mortality, and its main clinical value may lie in directing attention to the right heart—a hypothesis that requires external validation in independent AHF cohorts.

## Figures and Tables

**Table 1:** Valve involvement and individual lesion prevalence (n = 530). A valve was considered involved when carrying at least moderate stenosis and/or regurgitation. Italicized rows show individual lesion prevalence and are non-exclusive.

Finding	n (%)
Any valve involved (≥1)	378 (71.3%)
Mitral valve involved	235 (44.3%)
Tricuspid valve involved	232 (43.8%)
Aortic valve involved	146 (27.5%)
Multivalvular (≥2 valves)	185 (34.9%)
Moderate/severe mitral regurgitation	227 (42.8%)
Moderate/severe tricuspid regurgitation	232 (43.8%)
Moderate/severe aortic stenosis	105 (19.8%)
Moderate/severe aortic regurgitation	67 (12.6%)
Moderate/severe mitral stenosis	22 (4.2%)

**Table 2:** Baseline characteristics by multivalvular status. Continuous variables: median (IQR); categorical: n (%). Available denominators are shown where data were incomplete. CFS, Clinical Frailty Scale; CKD, chronic kidney disease; COPD, chronic obstructive pulmonary disease; eGFR, estimated glomerular filtration rate; E/e′, ratio of early mitral inflow to early diastolic mitral annular velocity; LAVi, left atrial volume index; LVEF, left ventricular ejection fraction; NT-proBNP, N-terminal pro–B-type natriuretic peptide; KDIGO, Kidney Disease: Improving Global Outcomes; NYHA, New York Heart Association; PASP, pulmonary artery systolic pressure; RV, right ventricular; TAPSE, tricuspid annular plane systolic excursion; TIA, transient ischemic attack. The *p* value for coronary artery disease refers to the three-category distribution and therefore reflects differences in ascertainment as well as in disease.

Characteristic	Multivalvular (n = 185)	≤1 Valve (n = 345)	*p*
**Demographics and heart-failure profile**			
Age, years	83 (78–87)	79 (73–85)	<0.001
Male sex	89 (48.1%)	195 (56.5%)	0.078
De novo heart failure	30 (16.2%)	116 (33.6%)	<0.001
Atrial fibrillation	124 (67.0%)	189 (54.8%)	0.008
Type of Atrial Fibrillation Paroxysmal	28 (15.1%)	73 (21.2%)	0.002
Persistent or Permanent	88 (47.6%)	96 (27.8%)	
Unknown	8 (4.3%)	20 (5.8%)	
Ischemic etiology	54 (29.2%)	113 (32.8%)	0.46
Frailty (CFS ≥ 5)	155 (83.8%)	197 (57.1%)	<0.001
Baseline NYHA class			<0.001
I	2 (1.1%)	20 (5.8%)	
II	17 (9.2%)	86 (24.9%)	
III	116 (62.7%)	205 (59.4%)	
IV	50 (27.0%)	34 (9.9%)	
**Comorbidities**			
Coronary artery disease			0.005
Documented	74 (40.0%)	164 (47.5%)	
Absent	33 (17.8%)	83 (24.1%)	
Not assessed	78 (42.2%)	98 (28.4%)	
Arterial hypertension	157 (84.9%)	305 (88.4%)	0.31
Diabetes mellitus	79 (42.7%)	196 (56.8%)	0.003
Chronic kidney disease	114 (61.6%)	160 (46.4%)	0.001
Airway disease (COPD)	47 (25.4%)	122 (35.4%)	0.025
Prior stroke/TIA	14 (7.6%)	25 (7.2%)	1.00
Active malignancy	19 (10.3%)	32 (9.3%)	0.83
Permanent pacemaker	16 (8.6%)	35 (10.1%)	0.69
Implantable cardioverter-defibrillator	8 (4.3%)	23 (6.7%)	0.37
Prior valve replacement (any)	10 (5.4%)	23 (6.7%)	0.70
**Laboratory and hemodynamics**			
NT-proBNP, pg/mL (n = 181/338)	7674 (4034–13,893)	4520 (2580–8944)	<0.001
Baseline creatinine, mg/dL	1.20 (0.90–1.50)	1.10 (0.90–1.40)	0.008
Baseline eGFR, mL/min/1.73 m^2^	54 (39–70)	64 (45–78)	<0.001
Admission eGFR, mL/min/1.73 m^2^	49 (35–67)	62 (43–78)	<0.001
**Baseline CKD stage (KDIGO)**			0.002
Stage 1 (eGFR ≥ 90)	8 (4.3%)	40 (11.6%)	
Stage 2 (60–89)	63 (34.1%)	145 (42.0%)	
Stage 3a (45–59)	50 (27.0%)	75 (21.7%)	
Stage 3b (30–44)	43 (23.2%)	66 (19.1%)	
Stage 4 (15–29)	21 (11.4%)	19 (5.5%)	
Right heart failure	112 (60.5%)	114 (33.0%)	<0.001
**Echocardiography**			
LVEF, %	45 (30–55)	50 (30–55)	0.38
LVEF < 35%	58 (31.4%)	108 (31.3%)	0.35
LVEF 35–39%	8 (4.3%)	24 (7.0%)	
LVEF 40–49%	29 (15.7%)	39 (11.3%)	
LVEF ≥ 50%	90 (48.6%)	174 (50.4%)	
E/e′ (n = 180/339)	20.0 (15–26)	15.5 (12–20)	<0.001
LAVi, mL/m^2^ (n = 184/345)	59.5 (50–73)	48.5 (41–58)	<0.001
TAPSE, mm (n = 184/345)	17.0 (14–18.5)	18.0 (16–20)	<0.001
RV S′, cm/s (n = 184/345)	9.5 (8–11)	11.0 (9–12)	<0.001
RV dilation	66 (35.7%)	64 (18.6%)	<0.001
PASP, mmHg (n = 184/345)	50 (44–60)	40 (30–45)	<0.001

**Table 3:** Medical therapy at admission and at discharge by multivalvular status. Values are n (%) or median (IQR). Discharge therapy is reported for the 503 patients who survived the index hospitalization. Guideline-directed medical therapy (GDMT) pillars counts renin–angiotensin system inhibition (ACE inhibitor, ARB or ARNI), beta-blocker, mineralocorticoid receptor antagonist and SGLT2 inhibitor, and is shown for patients with LVEF < 40%. ARB, angiotensin receptor blocker; ARNI, angiotensin receptor–neprilysin inhibitor; GDMT, guideline-directed medical therapy; HFrEF, heart failure with reduced ejection fraction; SGLT2, sodium–glucose cotransporter 2.

Therapy	Multivalvular (n = 185)	≤1 Valve (n = 345)	*p*
**At admission**			
ACE inhibitor or ARB	80 (43.2%)	187 (54.2%)	0.021
ARNI	8 (4.3%)	12 (3.5%)	0.80
Beta-blocker	112 (60.5%)	190 (55.1%)	0.26
Mineralocorticoid receptor antagonist	45 (24.3%)	69 (20.0%)	0.30
SGLT2 inhibitor	45 (24.3%)	63 (18.3%)	0.12
Furosemide	120 (64.9%)	145 (42.0%)	<0.001
Furosemide dose, mg	40 (0–40)	0 (0–40)	<0.001
**At discharge (n = 168 vs. 335)**			
ACE inhibitor or ARB	51 (30.4%)	146 (43.6%)	0.006
ARNI	12 (7.1%)	43 (12.8%)	0.075
Beta-blocker	124 (73.8%)	267 (79.7%)	0.17
Mineralocorticoid receptor antagonist	105 (62.5%)	188 (56.1%)	0.20
SGLT2 inhibitor	98 (58.3%)	225 (67.2%)	0.064
Furosemide	156 (92.9%)	290 (86.6%)	0.051
Furosemide dose, mg	40 (40–80)	40 (40–40)	<0.001
GDMT pillars at discharge, HFrEF (n = 56 vs. 126)	3 (1–3)	3 (2–4)	<0.001

**Table 4:** Patterns of valve involvement among patients with multivalvular disease (n = 185). Valve-level combinations are mutually exclusive and sum to 185. Lesion-level combinations are non-exclusive, as a patient may contribute to more than one pairing. Mortality estimates by specific valve combination are descriptive and were not intended for formal between-pattern comparison.

Pattern	n (% of Multivalvular)	1-Year Mortality
**Valve-level combinations**		
Mitral + tricuspid	89 (48.1%)	38.2%
All three valves	50 (27.0%)	54.0%
Aortic + mitral	30 (16.2%)	33.3%
Aortic + tricuspid	16 (8.6%)	31.2%
**Lesion-level combinations (non-exclusive)**		
Mitral regurgitation + tricuspid regurgitation	133 (71.9%)	—
Mitral regurgitation + aortic regurgitation	47 (25.4%)	—
Mitral regurgitation + aortic stenosis	46 (24.9%)	—
Tricuspid regurgitation + aortic stenosis	42 (22.7%)	—

**Table 5:** One-year all-cause mortality by number of involved valves.

Valves Involved	n	1-Year Mortality
0	152	19.7% (30/152)
1	193	24.9% (48/193)
2	135	36.3% (49/135)
3	50	54.0% (27/50)
Log-rank across groups		*p* < 0.001
Per additional valve (crude HR)		1.51 (1.28–1.78)

**Table 6:** Cox proportional-hazards models for one-year all-cause mortality (n = 519; 149 events). Adjusted models include admission log NT-proBNP, frailty (CFS ≥ 5) and age. Likelihood-ratio test comparing the categorical with the continuous parameterization: *p* = 0.71.

Model	HR (95% CI)	*p*
Per additional valve—crude	1.51 (1.28–1.78)	<0.001
Per additional valve—adjusted	1.22 (1.03–1.45)	0.023
Multivalvular (≥2) vs. ≤1—crude	2.07 (1.51–2.84)	<0.001
Multivalvular (≥2) vs. ≤1—adjusted	1.48 (1.06–2.07)	0.020
Categorical parameterization—adjusted		
1 valve vs. 0	1.02 (0.64–1.63)	0.94
2 valves vs. 0	1.41 (0.88–2.27)	0.15
3 valves vs. 0	1.69 (0.98–2.91)	0.060

**Table 7:** Renal characteristics and composite outcomes by number of involved valves. Continuous variables: median (IQR); categorical: n (%). Adjusted Cox models for both composite endpoints are presented in Supplementary Table S4. AHF, acute heart failure; CKD, chronic kidney disease; eGFR, estimated glomerular filtration rate; IQR, interquartile range; KDIGO, Kidney Disease: Improving Global Outcomes; RRT, renal replacement therapy.

Parameter	0 Valves (n = 152)	1 Valve (n = 193)	2 Valves (n = 135)	3 Valves (n = 50)	*p*
**Renal characteristics**					
Baseline eGFR, mL/min/1.73 m^2^	66 (47–82)	62 (44–77)	55 (40–72)	47 (37–66)	<0.001
CKD stage ≥ 3 (KDIGO)	66 (43.4%)	94 (48.7%)	80 (59.3%)	34 (68.0%)	0.004
In-hospital acute kidney injury	69 (45.4%)	93 (48.2%)	68 (50.4%)	25 (50.0%)	0.85
**One-year composite outcomes**					
Death or RRT	32 (21.1%)	50 (25.9%)	52 (38.5%)	30 (60.0%)	<0.001
Death, AHF rehospitalization or RRT	58 (38.2%)	86 (44.6%)	74 (54.8%)	40 (80.0%)	<0.001

**Table 8:** Sensitivity analyses: association of multivalvular disease with one-year mortality under alternative definitions. Adjusted models include admission log NT-proBNP, frailty (CFS ≥ 5) and age. Mortality is shown for patients meeting versus not meeting each definition.

Definition	n (%)	1-Year Mortality	Crude HR (95% CI)	Adjusted HR (95% CI)	*p*
≥2 valves, ≥moderate (primary)	185 (34.9%)	41.1% vs. 22.6%	2.07 (1.51–2.84)	1.48 (1.06–2.07)	0.020
≥2 valves, each with severe disease	39 (7.4%)	48.7% vs. 27.5%	2.01 (1.25–3.25)	1.43 (0.88–2.33)	0.15
≥1 severe + ≥1 other ≥ moderate	116 (21.9%)	46.6% vs. 24.2%	2.24 (1.61–3.13)	1.60 (1.14–2.25)	0.007
Left-sided: severe aortic or mitral + ≥moderate other left-sided valve	44 (8.3%)	59.1% vs. 26.3%	2.88 (1.88–4.39)	1.85 (1.20–2.86)	0.005

## Data Availability

The data supporting the findings of this study are not publicly available due to ethical considerations and patient confidentiality. Access to the dataset may be provided upon reasonable request to the corresponding author, subject to approval by the Scientific Council of Venizelio General Hospital of Heraklion.

## Supplementary Materials

The following supporting information can be downloaded at: https://www.mdpi.com/article/10.3390/medsci14050564/s1. Table S1: Operational definition of valve involvement; Table S2: Full multivariable Cox models for one-year all-cause mortality; Table S3: Test of linearity for the valve-count exposure; Table S4: Full multivariable Cox models for the composite endpoints; Table S5: Valve involvement according to baseline KDIGO CKD stage; Table S6: Individual valve involvement and severity-weighted score; Table S7: Expanded adjustment model for one-year all-cause mortality; Table S8: Sensitivity models adding right-sided variables to the primary mortality model; Table S9: Valve burden and one-year mortality stratified by clinically manifest right heart failure; Table S10: Distribution of lesion severity by valve, and severe lesions by multivalvular status; Table S11: Propensity-based and additional sensitivity analyses; Table S12: Post-discharge sensitivity analysis of valve burden and mortality among hospital survivors; Figure S1: Kaplan–Meier freedom from the cardiorenal composite endpoint by number of involved valves; Figure S2: Directed acyclic graph of the assumed relationships; Figure S3: Model-based adjusted survival by number of involved valves.

## Abbreviations

The following abbreviations are used in this manuscript:

ACE	Angiotensin-converting enzyme
AF	Atrial fibrillation
AHF	Acute heart failure
AKI	Acute kidney injury
ARB	Angiotensin receptor blocker
ARNI	Angiotensin receptor–neprilysin inhibitor
CFS	Clinical Frailty Scale
CI	Confidence interval
CKD	Chronic kidney disease
COPD	Chronic obstructive pulmonary disease
EACTS	European Association for Cardio-Thoracic Surgery
EACVI	European Association of Cardiovascular Imaging
eGFR	Estimated glomerular filtration rate
E/e′	Ratio of early mitral inflow to early diastolic mitral annular velocity
ERS	European Respiratory Society
ESC	European Society of Cardiology
ESC-EORP	European Society of Cardiology EURObservational Research Programme
GDMT	Guideline-directed medical therapy
HF	Heart failure
HFrEF	Heart failure with reduced ejection fraction
HR	Hazard ratio
IQR	Interquartile range
KDIGO	Kidney Disease: Improving Global Outcomes
LAVi	Left atrial volume index
LVEF	Left ventricular ejection fraction
NT-proBNP	N-terminal pro–B-type natriuretic peptide
NYHA	New York Heart Association
PASP	Pulmonary artery systolic pressure
RRT	Renal replacement therapy
RV	Right ventricular
SGLT2	Sodium–glucose cotransporter 2
TAPSE	Tricuspid annular plane systolic excursion
TIA	Transient ischemic attack
