# Supplementary material for: Multivalvular Involvement in Acute Heart Failure: Associations with One-Year Outcomes and the Right-Heart Phenotype
Source: Med Sci (Basel). 2026 Sep 12;14(5):564. doi: 10.3390/medsci14050564 (PMC13609675; doi:10.3390/medsci14050564)

Supplementary Material

Multivalvular Involvement in Acute Heart Failure: Associations with One-Year Outcomes and the Right-Heart Phenotype

Table S1. Operational definition of valve involvement used in the present analysis.

**Anatomical valve**	**Lesions considered**	**Threshold for "involvement"**
Aortic	Aortic stenosis; aortic regurgitation	At least moderate stenosis and/or regurgitation
Mitral	Mitral regurgitation; mitral stenosis	At least moderate stenosis and/or regurgitation
Tricuspid	Tricuspid regurgitation	At least moderate regurgitation
Pulmonary	*Not routinely quantified*	*Not included*

Each anatomical valve was treated as a single unit, so that mixed disease of one valve (for example combined moderate aortic stenosis and aortic regurgitation) counted as involvement of that valve only. The number of involved valves ranged from 0 to 3, and multivalvular disease was defined as involvement of two or more valves. In patients with a previously implanted prosthesis, the prosthetic valve was counted as involved only where prosthetic dysfunction of at least moderate degree was documented. Lesion severity was graded according to contemporary ESC/EACTS and EACVI recommendations.

Table S2. Full multivariable Cox models for one-year all-cause mortality (n=519; 149 events).

**Covariate**	**Model A: valve count**	**p**	**Model B: multivalvular**	**p**
Number of involved valves (per valve)	1.22 (1.03–1.45)	0.023	—	—
Multivalvular disease (≥2 valves)	—	—	1.48 (1.06–2.07)	0.020
Admission log NT-proBNP (per log-unit)	1.78 (1.47–2.15)	<0.001	1.80 (1.49–2.17)	<0.001
Frailty (CFS ≥5)	2.06 (1.20–3.55)	0.009	2.05 (1.19–3.54)	0.010
Age (per year)	1.02 (0.99–1.04)	0.126	1.02 (0.99–1.04)	0.124
Harrell's C-index	*0.714*		*0.713*	

Values are hazard ratios (95% confidence intervals). The proportional-hazards assumption was satisfied for every term (scaled Schoenfeld residuals: valve count p=0.84, log NT-proBNP p=0.12, frailty p=0.93, age p=0.76). CFS, Clinical Frailty Scale; NT-proBNP, N-terminal pro–B-type natriuretic peptide.

Table S3. Test of linearity for the valve-count exposure (one-year all-cause mortality).

**Parameterization**	**Crude HR (95% CI)**	**p**	**Adjusted HR (95% CI)**	**p**
Continuous (per additional valve)	1.51 (1.28–1.78)	<0.001	1.22 (1.03–1.45)	0.023
Categorical (reference = 0 valves)				
1 valve	1.34 (0.85–2.12)	0.203	1.02 (0.64–1.63)	0.944
2 valves	2.15 (1.36–3.38)	0.001	1.41 (0.88–2.27)	0.152
3 valves	3.35 (1.99–5.63)	<0.001	1.69 (0.98–2.91)	0.060

Adjusted models include admission log NT-proBNP, frailty (CFS ≥5) and age. A likelihood-ratio test comparing the adjusted categorical with the adjusted continuous model gave χ^2 = 0.69 on 2 degrees of freedom (p=0.71); adding a quadratic term gave no improvement in fit (p=0.63). There was therefore no evidence of departure from linearity, although no individual category reached significance against the reference and the adjusted gradient was driven predominantly by patients with two or three involved valves.

Table S4. Full multivariable Cox models for the composite endpoints (n=519).

**Covariate**	**Death or RRT (159 events)**	**p**	**Triple composite (253 events)**	**p**
Models with valve count				
Number of involved valves (per valve)	1.26 (1.06–1.48)	0.007	1.21 (1.06–1.39)	0.004
Admission log NT-proBNP (per log-unit)	1.75 (1.46–2.11)	<0.001	1.36 (1.18–1.57)	<0.001
Frailty (CFS ≥5)	2.29 (1.35–3.91)	0.002	2.60 (1.74–3.87)	<0.001
Age (per year)	1.01 (0.99–1.03)	0.357	1.00 (0.98–1.02)	0.907
Harrell's C-index	*0.717*		*0.675*	
Models with multivalvular disease as a dichotomy				
Multivalvular disease (≥2 valves)	1.55 (1.13–2.14)	0.007	1.39 (1.08–1.80)	0.011
Admission log NT-proBNP	1.78 (1.48–2.13)	<0.001	1.38 (1.20–1.59)	<0.001

(per log-unit)				
Frailty (CFS ≥5)	2.29 (1.34–3.91)	0.002	2.60 (1.74–3.89)	<0.001
Age (per year)	1.01 (0.99–1.04)	0.346	1.00 (0.98–1.02)	0.984
Harrell's C-index	*0.717*		*0.672*	

Values are hazard ratios (95% confidence intervals). Both composites were analysed as time to the first occurrence of any component over the full cohort; death was treated as a terminal event, and initiation of renal replacement therapy closed follow-up for the rehospitalization component. The death-or-RRT composite adds only 10 events beyond the 154 deaths and is therefore driven predominantly by mortality. RRT, renal replacement therapy.

Table S5. Valve involvement according to baseline KDIGO chronic kidney disease stage.

Baseline KDIGO stage	n	Any valve involved	Multivalvular (≥2 valves)	Mean no. of valves	Aortic	Mitral	Tricuspid
Stage 1 (eGFR ≥90)	48	58.3%	16.7%	0.77	14.6%	35.4%	27.1%
Stage 2 (60–89)	208	68.3%	30.3%	1.06	26.9%	39.9%	38.9%
Stage 3a (45–59)	125	75.2%	40.0%	1.23	24.0%	46.4%	52.8%
Stage 3b (30–44)	109	75.2%	39.4%	1.32	35.8%	48.6%	47.7%
Stage 4 (15–29)	40	80.0%	52.5%	1.45	35.0%	60.0%	50.0%
p across stages		*0.088*	*0.002*				

eGFR in mL/min/1.73m². The Spearman correlation between baseline eGFR and the number of involved valves, calculated across all 530 patients, was $\varrho = -0.18$ (p<0.001); it is not a test for trend in the mean valve count across the categories shown above. Adjusted for age, sex and atrial fibrillation, the odds ratio for multivalvular disease in KDIGO stage ≥3 versus stages 1–2 was 1.45 (95% CI 0.98–2.12, p=0.06).

Table S6. Individual valve involvement and severity-weighted score: adjusted models for one-year mortality (n=519; 149 events).

Model / covariate	HR (95% CI)	p	C-index
Model C: all three valves entered simultaneously			*0.714*

Aortic valve involved	1.12 (0.80–1.59)	0.506	
Mitral valve involved	1.35 (0.95–1.91)	0.094	
Tricuspid valve involved	1.16 (0.83–1.64)	0.383	
Model D: severity-weighted score			*0.715*
Severity-weighted valve score (per point, range 0–6)	1.15 (1.04–1.28)	0.009	
Reference: simple valve count	*1.22 (1.03–1.45)*	*0.023*	*0.714*

All models adjusted for admission log NT-proBNP, frailty (CFS ≥5) and age. The severity-weighted score sums the severity grade of each anatomical valve (0 absent or mild, 1 moderate, 2 severe; range 0–6), taking the higher of the two lesion grades where a valve carried both stenosis and regurgitation.

Table S7. Expanded adjustment model for one-year all-cause mortality (n=519; 149 events).

**Covariate**	**Model E: valve count**	**p**	**Model F: multivalvular**	**p**
Number of involved valves (per valve)	1.19 (1.01–1.42)	0.043	—	—
Multivalvular disease (≥2 valves)	—	—	1.44 (1.03–2.01)	0.033
Admission log NT-proBNP (per log-unit)	1.81 (1.43–2.28)	<0.001	1.84 (1.46–2.32)	<0.001
Frailty (CFS ≥5)	1.73 (0.99–3.02)	0.056	1.72 (0.98–3.00)	0.058
Age (per year)	1.02 (1.00–1.05)	0.110	1.02 (1.00–1.05)	0.105
Male sex	1.26 (0.88–1.81)	0.208	1.24 (0.86–1.78)	0.249
Atrial fibrillation	1.07 (0.76–1.50)	0.695	1.08 (0.77–1.52)	0.653
LVEF (per %)	1.01 (0.99–1.02)	0.417	1.01 (0.99–1.02)	0.428
Baseline eGFR (per mL/min/1.73 m^2)	1.00 (0.99–1.01)	0.859	1.00 (0.99–1.01)	0.793
De novo heart failure	0.49 (0.29–0.82)	0.007	0.48 (0.29–0.81)	0.006
Harrell's C-index	*0.728*		*0.728*	

Values are hazard ratios (95% confidence intervals). Right-sided variables were deliberately not entered here and are examined separately in Table S8, since they are strongly intercorrelated and plausibly lie on the causal pathway between tricuspid involvement and outcome. eGFR, estimated glomerular filtration rate; LVEF, left ventricular ejection fraction.

Table S8. Sensitivity models adding right-sided variables to the primary mortality model (n≥517; 149 events).

Model	Per additional valve, HR (95% CI)	p	Multivalvular ≥2, HR (95% CI)	p
Primary model (reference)	1.22 (1.03–1.45)	0.023	1.48 (1.06–2.07)	0.020
+ Pulmonary artery systolic pressure	1.18 (0.98–1.42)	0.083	—	—
+ TAPSE	1.18 (0.99–1.41)	0.058	—	—
+ Tricuspid annular S′	1.19 (1.00–1.42)	0.051	—	—
+ Right ventricular dilation	1.18 (0.99–1.41)	0.065	—	—
+ Right heart failure (clinical)	1.15 (0.96–1.37)	0.130	1.33 (0.94–1.87)	0.103
+ All four right-sided terms	1.13 (0.94–1.37)	0.195	1.29 (0.91–1.85)	0.154

All models additionally adjusted for admission log NT-proBNP, frailty (CFS ≥5) and age. Right-sided echocardiographic measures were entered individually because they are strongly intercorrelated. Because right-sided involvement is a downstream consequence of significant tricuspid regurgitation, these models constitute adjustment for a plausible intermediate and the resulting attenuation should be interpreted accordingly. The same adjustments applied to the composite endpoints gave closely similar point estimates: after adjustment for clinically manifest right heart failure the hazard ratio per additional valve was 1.19 (1.00–1.41, p=0.051) for death or renal replacement therapy and 1.19 (1.03–1.36, p=0.015) for the triple composite. TAPSE, tricuspid annular plane systolic excursion.

Table S9. Valve burden and one-year mortality stratified by clinically manifest right heart failure.

Stratum	n	Deaths	Crude HR per valve (95% CI)	p	Adjusted HR per valve (95% CI)	p
Without right heart failure	304	66 (21.7%)	1.25 (0.96–1.63)	0.102	1.06 (0.80–1.41)	0.693

With right heart failure	226	88 (38.9%)	1.52 (1.22–1.90)	<0.001	1.22 (0.97–1.55)	0.092

Adjusted models include admission log NT-proBNP, frailty (CFS ≥5) and age. One-year mortality across zero, one, two and three involved valves was 20.7%, 19.2%, 21.4% and 47.1% in the absence of right heart failure and 17.1%, 34.2%, 46.8% and 57.6% in its presence; the prevalence of right heart failure itself rose from 27.0% to 37.8%, 58.5% and 66.0% across the same strata. The formal interaction between valve burden and right heart failure was not significant (p=0.45) and the stratum without right heart failure contained only 66 deaths, so this analysis is underpowered for effect modification and is hypothesis-generating. The same stratified pattern was observed for the triple composite endpoint: the adjusted hazard ratio per additional valve was 1.09 (0.89–1.34, p=0.38; 122 events) without right heart failure and 1.29 (1.07–1.56, p=0.008; 131 events) with it (interaction p=0.41).

Table S10. Distribution of lesion severity by valve, and severe lesions by multivalvular status (n=530).

**Valve / lesion**	**None or mild**	**Moderate**	**Severe**
Aortic stenosis	425 (80.2%)	40 (7.5%)	*65 (12.3%)*
Aortic regurgitation	463 (87.4%)	60 (11.3%)	*7 (1.3%)*
Mitral regurgitation	303 (57.2%)	175 (33.0%)	*52 (9.8%)*
Mitral stenosis	508 (95.8%)	17 (3.2%)	*5 (0.9%)*
Tricuspid regurgitation	298 (56.2%)	128 (24.2%)	*104 (19.6%)*
Severe lesion, by group	Multivalvular (n=185)	≤1 valve (n=345)	*p*
Severe aortic stenosis	37 (20.0%)	28 (8.1%)	*<0.001*
Severe aortic regurgitation	7 (3.8%)	0 (0.0%)	*0.001*
Severe mitral regurgitation	43 (23.2%)	9 (2.6%)	*<0.001*
Severe mitral stenosis	4 (2.2%)	1 (0.3%)	*0.053*
Severe tricuspid regurgitation	70 (37.8%)	34 (9.9%)	*<0.001*
Any severe lesion	116 (62.7%)	72 (20.9%)	*<0.001*
Severe aortic stenosis by valve count	0 valves 0%; 1 valve 14.5%	2 valves 11.9%; 3 valves 42.0%	*<0.001*

Values are n (%). Percentages in the upper block are of the whole cohort; those in the lower block are within each group.

Table S11. Propensity-based and additional sensitivity analyses for one-year all-cause mortality (n=519; 149 events).

Analysis	Per additional valve, HR (95% CI)	p	Multivalvular ≥2, HR (95% CI)	p
Primary model (reference)	1.22 (1.03–1.45)	0.023	1.48 (1.06–2.07)	0.020
+ Severe aortic stenosis	1.18 (0.99–1.41)	0.060	1.44 (1.03–2.01)	0.034
+ Disease-modifying agents at admission	1.22 (1.03–1.45)	0.023	1.48 (1.06–2.06)	0.022
Propensity score as covariate	1.15 (0.96–1.37)	0.128	1.33 (0.94–1.87)	0.104
Stabilized inverse-probability weighting	—	—	1.31 (0.92–1.86)	0.132
Propensity score excluding right heart failure	1.19 (1.00–1.41)	0.050	1.43 (1.02–2.00)	0.036
Inverse-probability weighting, model excluding right heart failure	—	—	1.40 (0.99–1.98)	0.055

Models in the first three rows are additionally adjusted for admission log NT-proBNP, frailty (CFS ≥5) and age. The propensity score for multivalvular involvement was estimated by logistic regression on age, sex, atrial fibrillation, LVEF, baseline eGFR, de novo status, frailty, log NT-proBNP, chronic kidney disease and right heart failure (C-statistic 0.738); weights were stabilized and trimmed at the 99th percentile. A second propensity model was fitted omitting clinically manifest right heart failure (C-statistic 0.718), because right heart failure may act as an intermediate rather than a conventional confounder; the association was retained in that model. These are exploratory sensitivity analyses and were not corrected for multiplicity.

Table S12. Post-discharge sensitivity analysis of valve burden and mortality among hospital survivors, with time zero at discharge.

Model	Per additional valve, HR (95% CI)	p	Multivalvular ≥2, HR (95% CI)	p
Crude	1.45 (1.21–1.73)	<0.001	1.89 (1.33–2.68)	<0.001
Adjusted	1.17 (0.97–1.41)	0.094	1.35 (0.94–1.94)	0.110

Restricted to the 503 patients who survived the index hospitalization; n=494 with complete covariates, 124 deaths. Adjusted models include admission log NT-proBNP,

frailty (CFS ≥5) and age. Time zero is the date of discharge. Because follow-up ended 12 months after admission, the post-discharge observation period varied according to length of stay, and survivors were censored at their individual end of follow-up. The 27 in-hospital deaths are excluded by design, which reduces the number of events from 149 to 124. The adjusted estimates remained directionally consistent with, but were attenuated relative to, the admission-anchored primary analysis.

Figure S1. Kaplan–Meier freedom from the cardiorenal composite endpoint (death or renal replacement therapy) according to the number of involved valves, over 12 months of follow-up. Event-free survival diverges progressively with increasing valve burden (log-rank p<0.001). Group labels correspond to those used in main-text Figure 1.

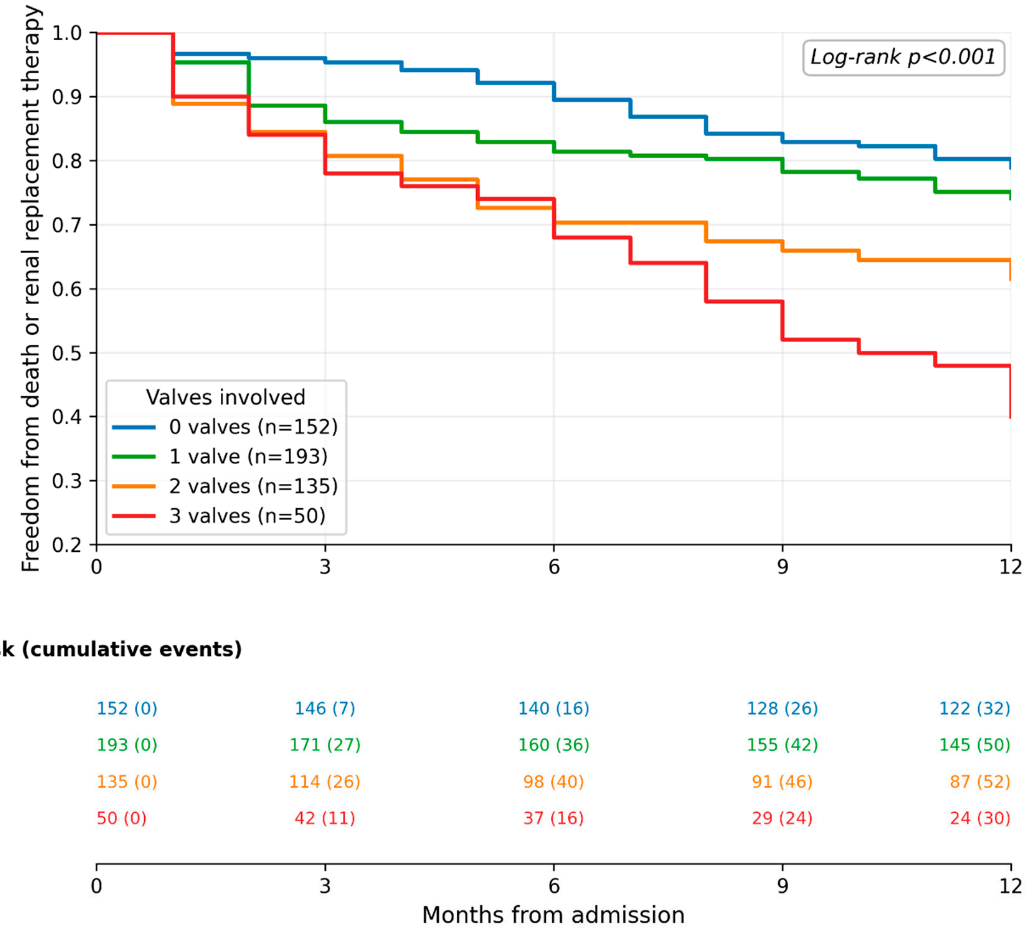

Figure S2. Directed acyclic graph of the assumed relationships between multivalvular valve burden, the intermediate variables and one-year mortality. Solid arrows denote the hypothesized causal pathways: valve burden is assumed to act on outcome through increased cardiac load and reduced physiological reserve (indexed by NT-proBNP and the Clinical Frailty Scale) and through right-sided involvement (pulmonary artery systolic pressure, right ventricular function and clinically manifest right heart failure). Dashed arrows denote a shared substrate — age, atrial fibrillation and chronic kidney disease — acting as a common cause of the exposure, of both intermediates and of the outcome, and therefore representing potential confounding. Under this structure the crude analysis is closest to the total observed association; the primary model, which conditions on NT-proBNP, frailty and age, estimates a partly conditional association; and models that additionally include right-sided variables condition on plausible intermediates, so that attenuation in those models cannot be interpreted as evidence against an independent effect. CFS, Clinical Frailty Scale; NT-proBNP, N-terminal pro–B-type natriuretic peptide; PASP, pulmonary artery systolic pressure; RV, right ventricular.

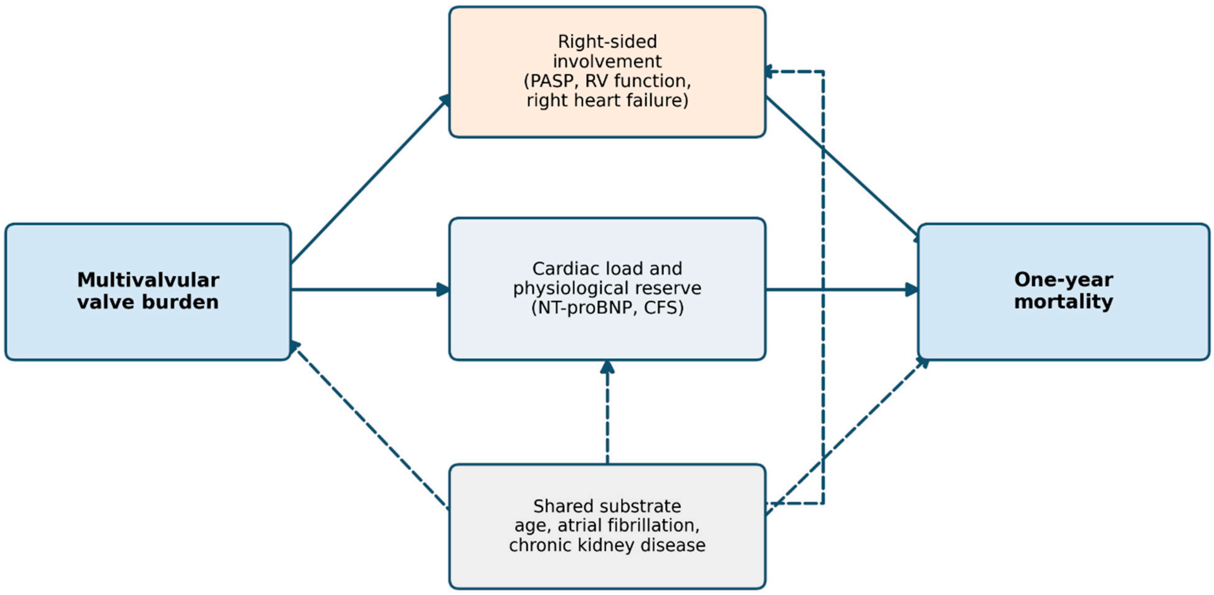

Figure S3. Model-based adjusted survival by number of involved valves, derived from the primary Cox model at mean covariate values (admission log NT-proBNP, frailty [CFS ≥5] and age; n=519, 149 events). Adjusted survival at 12 months was 81.2%, 77.5%, 73.3% and 68.5% for zero, one, two and three involved valves respectively. The ordering matches that of the unadjusted Kaplan–Meier curves in main-text Figure 1, with attenuated separation once baseline differences in age, frailty and natriuretic peptide concentration are taken into account. Group labels correspond to those used in main-text Figure 1. CFS, Clinical Frailty Scale; NT-proBNP, N-terminal pro–B-type natriuretic peptide.

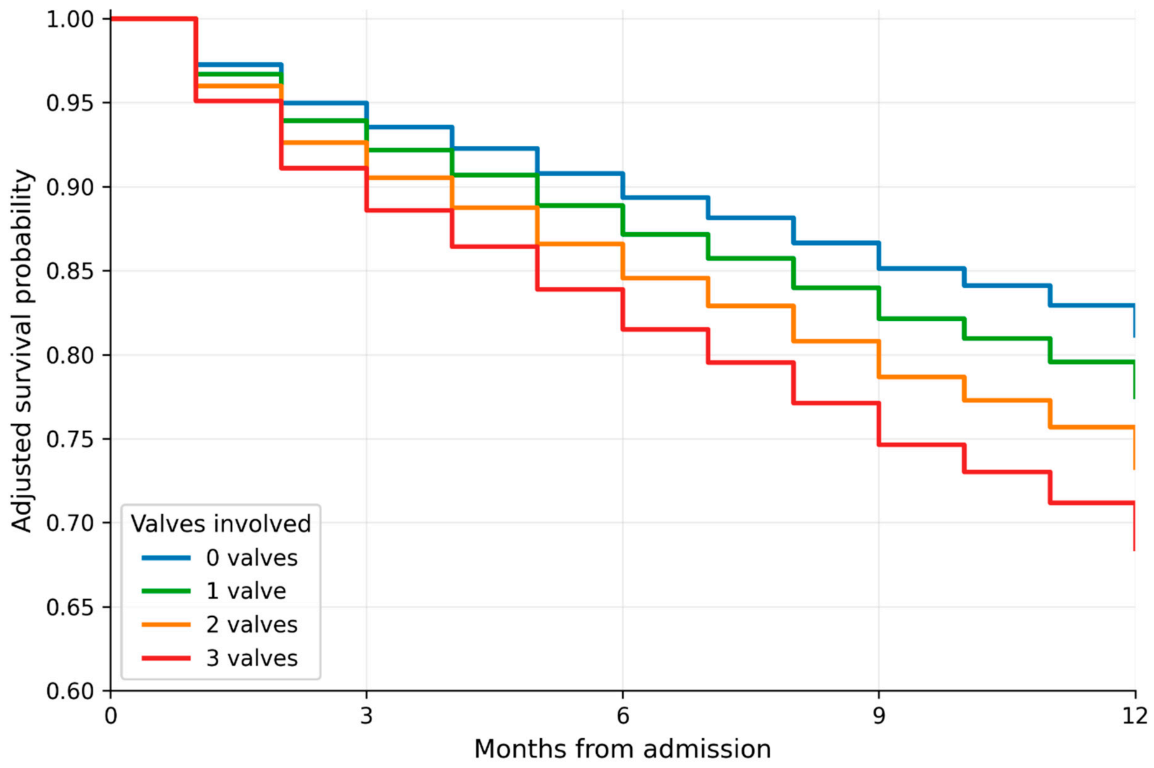

Supplement: Supplementary file 1 [file medsci-14-00564-s001.zip › Supplementary_CLEAN.pdf]
